# Temporal trends in cancer mortality attributable to high BMI in Asia: an age-period-cohort analysis based on GBD 1992–2021

**DOI:** 10.3389/fendo.2025.1691487

**Published:** 2025-10-10

**Authors:** Minguang Huang, Liejiong Wang, Ying Lou, Zhaoqi Qiu, Shengjian Yu, Feng Xuan

**Affiliations:** ^1^ Department of Radiation Oncology, Zhuji Affiliated Hospital of Wenzhou Medical University, Shaoxing, China; ^2^ Department of Medical Oncology, Zhuji Affiliated Hospital of Wenzhou Medical University, Shaoxing, China

**Keywords:** global burden of disease study, cancer, high BMI, Asia, age-period-cohort effect, decomposition analysis

## Abstract

**Background:**

High body mass index (BMI) is a well-established modifiable risk factor for cancer. This study aims to assess temporal trends and contributing factors in cancer mortality attributable to high BMI in Asia from 1992 to 2021.

**Methods:**

Data were obtained from the Global Burden of Disease (GBD) study. We analyzed mortality attributable to high BMI for total cancer and 11 specific subtypes across 34 Asian countries and five GBD regions from 1992 to 2021. Age-standardized mortality rates (ASMRs), average annual percentage change (AAPC), and age-period-cohort effects were calculated. Decomposition analysis quantified the contributions of aging, population growth, and epidemiological change to the changes in cancer deaths.

**Result:**

From 1992 to 2021, high BMI-related cancer deaths in Asia rose from 29,908 to 123,693, with ASMR increasing from 1.42 to 2.46 per 100,000 (AAPC: 1.92%, 95% CI: 1.85–1.98). South Asia experienced the most rapid ASMR growth (AAPC: 3.16%), while High-income Asia Pacific showed a decline after 1997. Sex-specific analyses revealed that colorectal, liver, and leukemia in males and colorectal, breast, and uterine cancers in females contributed most to ASMRs. Decomposition analysis indicated that the change in deaths was mainly driven by epidemiological change (118.20%), followed by population growth (115.54%) and aging (79.83%). Age-period-cohort analysis demonstrated rising mortality risks in successive birth cohorts, particularly in East and South Asia, while period and cohort effects declined in High-income Asia Pacific. Local drift analyses showed increasing mortality trends in older age groups across most regions.

**Conclusion:**

The burden of cancer ASMR attributable to high BMI in Asia has risen substantially over the past three decades. Substantial heterogeneity exists across regions, countries, sexes, and cancer types. Targeted prevention strategies and cancer control policies are urgently needed to address this rising public health challenge.

## 1 Introduction

High body mass index (BMI), encompassing overweight and obesity, is a major modifiable risk factor for non-communicable diseases, including cardiovascular disease, type 2 diabetes, and multiple cancers (1, 2). Since 1980, obesity prevalence has more than doubled in over 70 countries, reaching 603.7 million obese adults by 2015 (3). Recent global assessments estimated that high BMI contributed to 4–5% of all cancer deaths in 2019, equivalent to approximately 462,000 deaths, with an age-standardized mortality rate (ASMR) rising by 0.48% annually in recent decades (4, 5). Epidemiological studies have linked high BMI to increased risk for multiple cancers, including those of the breast, colorectum, liver, kidney, ovary, and thyroid, as well as multiple myeloma and others (2, 6). Mechanistically, excess adiposity promotes chronic inflammation, hormonal dysregulation, and immune impairment, fostering a pro-carcinogenic environment (7).

Asia is home to over 60% of the world’s population and is undergoing rapid demographic and lifestyle changes that predispose to both obesity and cancer (8, 9). Economic growth, urbanization, and globalization across Asian countries have led to increasingly sedentary lifestyles and adoption of high-energy “Western” diets, resulting in a sharp rise in overweight and obesity rates. While many Asian populations historically had relatively low obesity rates, this is no longer the case. For example, the prevalence of high BMI in parts of East and South Asia has been climbing steadily over the past three decades (10). Cancer already represents a leading cause of death in Asia, and nearly half of the global cancer cases occur in this region (11). The rising prevalence of obesity is poised to further exacerbate Asia’s cancer burden, creating a dual public health challenge.

Despite the evident importance of high BMI as a risk factor for cancer in Asia, detailed research in this context is limited. Most prior studies have examined the high BMI-related cancer burden at the global level or have focused on individual cancer types or specific countries, rather than analyzing Asia as a whole. For example, earlier global analyses quantified the overall burden of cancers attributable to high BMI, but they did not provide a detailed analysis for Asia specifically (4, 5). Other investigations have concentrated on single cancer sites (such as breast or colorectal cancer) (12, 13). Moreover, the age-period-cohort analysis is a powerful approach to disentangle whether temporal trends are primarily driven by population aging, by secular changes such as economic development and healthcare improvements, or by generational risk exposures such as increasing obesity in younger cohorts (14–16). However, previous studies based on age-period-cohort models in Asia were also largely focusing on single cancers (e.g., cervical cancer in Korea, ovarian cancer in China) or on mortality for broad all-site cancer categories in specific countries (e.g., Japan) (17–19).

In this research, we aim to addresses this gap by providing a pan-Asian analysis of cancer mortality attributable to high BMI across 34 countries and territories from 1992 to 2021. we examined total cancer and 11 major high-BMI-related cancers, including ovarian, breast, uterine, thyroid, kidney, colon and rectum, gallbladder and biliary tract, liver, leukemia, multiple myeloma, and non-Hodgkin lymphoma. An age-period-cohort framework was applied to distinguish the contributions of aging, temporal periods, and birth cohort effects on the observed mortality trends. The study findings may aid health authorities and policymakers in Asia in effectively allocating resources and implementing tailored interventions, including obesity prevention programs and cancer screening and treatment strategies, to reduce the growing impact of high BMI on cancer outcomes.

## 2 Methods

### 2.1 Data sources

The GBD 2021 study, a comprehensive and collaborative effort coordinated by the Institute for Health Metrics and Evaluation, provides estimates for 371 diseases and injuries, 88 risk factors, and covers 204 countries and territories (20, 21). It employs a variety of data sources, including vital registration systems, verbal autopsies, and surveillance systems, to generate robust estimates of disease burden. It also uses advanced statistical models to ensure the accuracy and reliability of the estimates, including the Cause of Death Ensemble model and the Bayesian meta-regression tool DisMod-MR 2.1. The attribution of cancer mortality to high BMI in this study is directly derived from the GBD 2021 comparative risk assessment framework. This framework estimates the burden of disease attributable to individual risk factors (including high BMI) using a counterfactual approach, which compares observed outcomes to a theoretical minimum-risk exposure distribution. Detailed analytical frameworks and calculation methodologies of the GBD study can be found in previous publications (20, 21). For this secondary analysis, we extracted age-, sex-, location-, and cancer-specific estimates of cancer mortality attributable to high BMI between 1992 and 2021 from the publicly available Global Health Data Exchange (http://ghdx.healthdata.org/gbd-results-tool). The data were stratified by age, sex, region, country, and cancer type. Individuals under 20 years of age were not included in the analysis due to their low mortality.

### 2.2 Definitions

Following the GBD 2021 study framework, we obtained data on the following 11 cancer subtypes: ovarian cancer, breast cancer, uterine cancer, thyroid cancer, kidney cancer, leukemia, multiple myeloma, non-Hodgkin lymphoma, colon and rectum cancer, gallbladder and biliary tract cancer, and liver cancer (21). The GBD study defines high BMI-attributable cancer burden only for cancer sites where there is sufficient evidence of a causal association between elevated BMI and cancer risk, as determined through systematic reviews and meta-analyses conducted by the GBD Risk Factors Collaborators (20). Each subtype was identified using the International Statistical Classification of Diseases and Related Health Problems, 10th Revision (ICD-10) and 9th Revision (ICD-9) codes (**Supplementary Table S1**). High BMI for adults (aged ≥20 years) was defined as ≥25 kg/m², following the GBD 2021 definition (20). Furthermore, 34 Asian countries and territories were classified into five GBD regions: High-income Asia Pacific (HIAP), East Asia, South Asia, Central Asia, and Southeast Asia (**Supplementary Table S2**) (22).

### 2.3 Statistical analysis

#### 2.3.1 Joinpoint regression analysis

We employed Joinpoint Regression software (version 5.1.0.0, National Cancer Institute, Bethesda, America) to assess temporal trends in ASMR of cancer attributable to high BMI in Asia from 1992 to 2021 (23). This method identifies statistically significant changes in trend by connecting a series of linear segments on a logarithmic scale, with each “joinpoint” indicating a significant shift in the slope of the trend. Log-linear models connected by joinpoints were fitted, starting from 0 joinpoints (a single line). Model building proceeded via a grid search with Monte Carlo permutation testing to determine whether additional joinpoints improved fit. To balance fit and interpretability and to avoid overfitting, the maximum number of joinpoints was capped at five. For each segment, we estimated the annual percentage change (APC) and 95% confidence interval (CI). The overall trend was summarized by the average annual percentage change (AAPC), computed as the length-weighted mean of segment-specific APCs. AAPC estimates with 95% CIs entirely above zero were considered indicative of an increasing trend, those entirely below zero as decreasing, and those including zero as stable.

#### 2.3.2 Decomposition analysis

We applied the decomposition analysis developed by Das Gupta to quantify the relative contributions of population growth, population ageing, and epidemiological change to temporal variations in cancer mortality attributable to high BMI in Asia between 1992 and 2021 (24). This approach mathematically isolates the effect of each factor while holding the others constant, enabling estimation of its proportional contribution to the overall change in mortality burden. Epidemiological change was defined as the variation in age-specific mortality rates after adjustment for changes in both population size and age structure, reflecting shifts in risk factor exposure, diagnostic practices, and treatment effectiveness. For each year from 1993 to 2021, we computed the percentage contribution of each determinant to the total change relative to the baseline year (1992).

#### 2.3.3 Age-period-cohort analysis

We applied an age-period-cohort framework to disentangle the independent effects of age, calendar period, and birth cohort on ASMR of cancer attributable to high BMI in Asia between 1992 and 2021 (14, 25). Age was grouped into successive 5-year intervals from 20–24 years to 95 plus years, and the study period was divided into six consecutive 5-year intervals (1992–1996, 1997–2001, 2002–2006, 2007–2011, 2012–2016, and 2017–2021). Birth cohorts were defined by subtracting age from the period midpoint, resulting in 21 partially overlapping cohorts from 1892–1901 to 1992–2001. The birth cohort 1967–1976 and the period 1992–1996 were selected as reference categories. It is important to note that while the choice of reference groups can influence the absolute values of the estimated effects, it does not affect the interpretation of the relative differences between groups. This is because the APC model estimates the effects of age, period, and cohort relative to the chosen reference groups. Therefore, the selection of reference groups is arbitrary and does not fundamentally alter the substantive conclusions of the study (16, 26).

However, the perfect linear relationship between age, period, and cohort (age = period – birth cohort) presents an identifiability problem, complicating the statistical estimation of their individual influences. In this study, we utilized the Age-Period-Cohort Network tool provided by the National Cancer Institute (https://analysistools.cancer.gov/apc/) to address the inherent identifiability issues more effectively through the application of a log-linear Poisson regression model (14, 27). Consequently, four key parameters were estimated: (i) local drift, the annual percentage change in age-specific mortality rates for each age group; (ii) longitudinal age curve, the fitted age-specific mortality rates in the reference cohort adjusted for period deviations, representing the age effect; (iii) period rate ratio (RR), the mortality rate in each period relative to the reference period, representing the period effect; and (iv) cohort RR, the mortality rate in each cohort relative to the reference cohort, representing the cohort effect. Statistical significance was assessed using the Wald χ² test, with a two-sided *p* value <0.05 considered statistically significant.

All data processing, analysis, and visualization were executed using R (version 4.4.1). A two-tailed p-value < 0.05 was used to establish statistical significance.

### 2.4 Ethics statement

Given the de-identified nature and public availability of the GBD data, this study is exempt from institutional ethical board review.

## 3 Result

### 3.1 Epidemiological characteristics of total cancer and its subtypes

**Figure 1:**
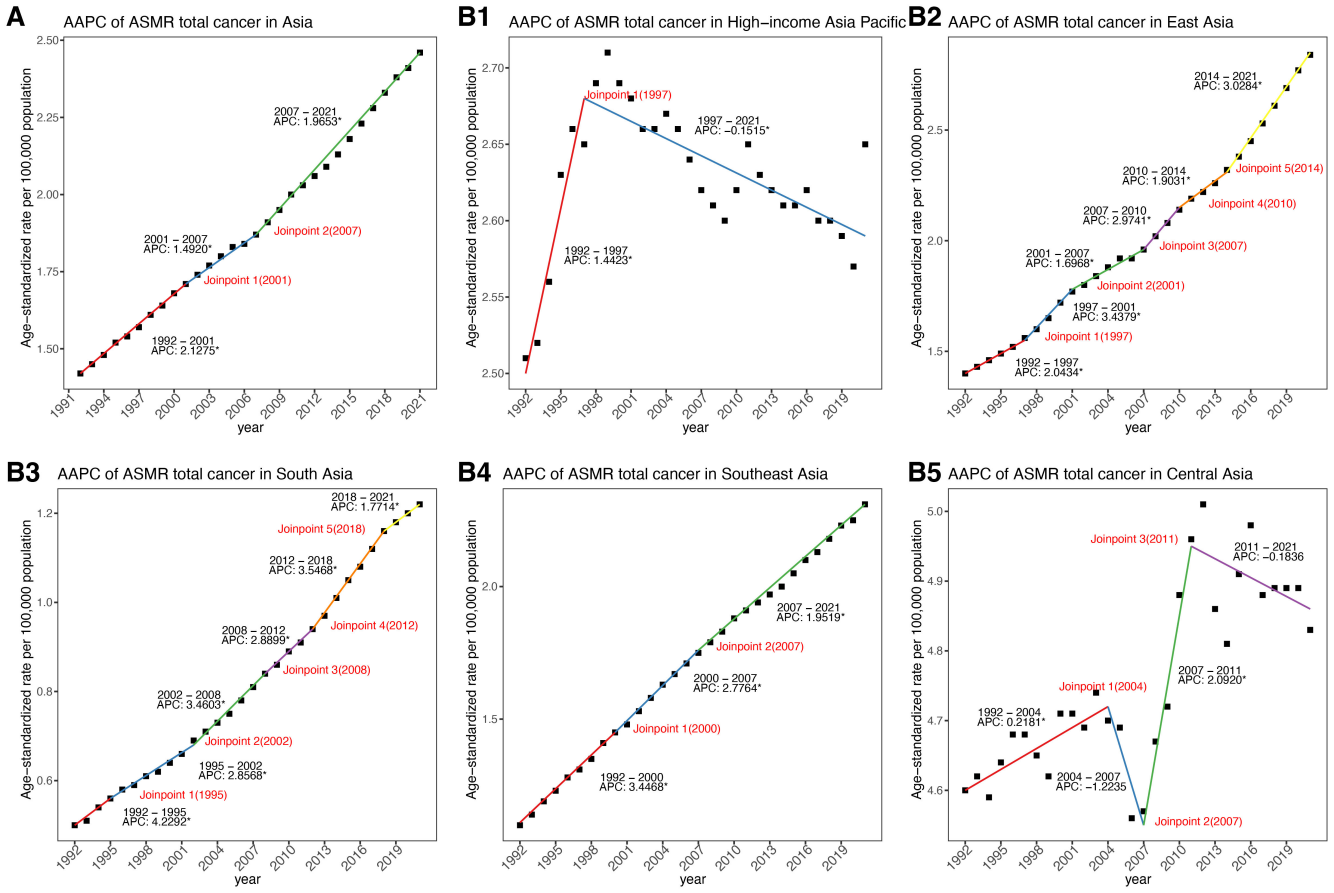
APCs and AAPC of ASMR in Asia and five GBD regions from 1992 to 2021. **(A)** In Asia. **(B)** Across five GBD regions. APC, annual percentage change; AAPC, average annual percentage change; ASMR, age–standardized mortality rate.

In 2021, a total of 123,693 cancer-related deaths (95% UI: 53,051 to 199,836) were recorded in Asia, compared with 29,908 deaths (95% UI: 15,177 to 46,527) in 1992 (**Table 1**). The ASMR increased from 1.42 per 100,000 population (95% UI: 0.71 to 2.21) in 1992 to 2.46 per 100,000 (95% UI: 1.06 to 3.98) in 2021, with an AAPC of 1.92% (95% CI: 1.85 to 1.98) (**Table 1**, **Figures 1A**).

**Table 1:** Trends in mortality of cancer attributable to high BMI in Asia from 1992 to 2021.

Location	Number of mortality (95%UI)		ASMR (per 100,000 population,95%UI)		Trend
	In 1992	In 2021	In 1992	In 2021	AAPC (95%CI, %)
Asia	29,908 (15,177 to 46,527)	123,693 (53,051 to 199,836)	1.42(0.71 to 2.21)	2.46(1.06 to 3.98)	1.92(1.85 to 1.98)
Male	11,952 (6,394 to 18,515)	51,980 (24,642 to 84,232)	1.13(0.61 to 1.77)	2.21(1.05 to 3.55)	2.33(2.19 to 2.48)
Female	17,956 (8,683 to 28,466)	71,712 (29,272 to 121,086)	1.67(0.80 to 2.66)	2.70(1.11 to 4.55)	1.68(1.62 to 1.73)
High-income Asia Pacific	5,335 (2,788 to 8,304)	13,084 (5,858 to 20,819)	2.51(1.31 to 3.89)	2.65(1.15 to 4.25)	0.12(0.04 to 0.21)
East Asia	13,058 (6,472 to 20,623)	61,372 (25,647 to 104,532)	1.40(0.68 to 2.24)	2.84(1.21 to 4.81)	2.48(2.25 to 2.70)
South Asia	3,053 (1,614 to 4,566)	18,111 (8,235 to 28,683)	0.50(0.26 to 0.75)	1.22(0.55 to 1.93)	3.16(2.99 to 3.32)
Central Asia	2,250 (1,009 to 3,635)	3,992 (1,691 to 6,589)	4.60(2.06 to 7.43)	4.83(2.04 to 8.00)	0.19(-0.14 to 0.51)
Southeast Asia	3,198 (1,584 to 4,995)	15,689 (6,553 to 25,864)	1.10(0.54 to 1.74)	2.31(0.97 to 3.81)	2.56(2.49 to 2.63)
Nepal	40 (22 to 64)	205 (101 to 340)	0.38(0.21 to 0.59)	0.84(0.41 to 1.39)	2.81(2.71 to 2.91)
Pakistan	782 (391 to 1,241)	3,797 (1,665 to 6,044)	1.34(0.66 to 2.15)	3.13(1.32 to 5.08)	2.97(2.87 to 3.07)
Bhutan	4 (2 to 6)	12 (5 to 22)	1.25(0.58 to 2.11)	1.90(0.83 to 3.52)	1.48(1.39 to 1.57)
Cambodia	52 (25 to 83)	224 (92 to 374)	1.01(0.50 to 1.62)	1.69(0.69 to 2.82)	1.81(1.76 to 1.86)
Bangladesh	150 (95 to 219)	918 (436 to 1,542)	0.30(0.18 to 0.45)	0.65(0.31 to 1.10)	2.75(2.41 to 3.09)
Lao People’s Democratic Republic	27 (12 to 43)	98 (40 to 165)	1.16(0.54 to 1.88)	1.96(0.80 to 3.31)	1.84(1.80 to 1.88)
Timor-Leste	1 (1 to 2)	6 (3 to 10)	0.38(0.20 to 0.64)	0.69(0.31 to 1.15)	2.10(1.84 to 2.36)
Myanmar	325 (151 to 549)	870 (363 to 1,533)	1.22(0.56 to 2.05)	1.67(0.70 to 2.92)	1.08(1.02 to 1.13)
Tajikistan	95 (39 to 150)	170 (74 to 282)	3.34(1.36 to 5.36)	2.81(1.21 to 4.70)	-0.61(-0.89 to -0.33)
Democratic People’s Republic of Korea	156 (74 to 266)	547 (244 to 961)	0.95(0.45 to 1.63)	1.72(0.76 to 3.04)	2.07(2.03 to 2.11)
Maldives	1 (1 to 2)	5 (2 to 9)	1.11(0.49 to 1.89)	1.3(0.52 to 2.28)	0.55(0.36 to 0.74)
India	2,077 (1,107 to 3,160)	13,180 (5,945 to 20,947)	0.41(0.21 to 0.63)	1.10(0.5 to 1.76)	3.48(3.27 to 3.69)
Kyrgyzstan	140 (60 to 231)	219 (90 to 352)	4.51(1.92 to 7.47)	4.43(1.80 to 7.12)	0.05(-0.30 to 0.40)
Mongolia	99 (42 to 171)	290 (118 to 487)	9.08(3.86 to 15.76)	12.56(5.08 to 21.01)	1.13(0.68 to 1.59)
Viet Nam	174 (90 to 278)	1,126 (468 to 1,891)	0.41(0.21 to 0.66)	1.08(0.45 to 1.81)	3.42(3.34 to 3.50)
Philippines	512 (205 to 833)	2,444 (962 to 4,070)	1.48(0.58 to 2.43)	2.81(1.09 to 4.69)	2.22(2.11 to 2.32)
Uzbekistan	324 (138 to 522)	890 (367 to 1,500)	2.66(1.11 to 4.33)	3.21(1.30 to 5.45)	0.64(0.15 to 1.12)
Indonesia	890 (447 to 1,455)	4,811 (1,903 to 8,348)	0.77(0.38 to 1.27)	1.84(0.73 to 3.20)	3.07(3.03 to 3.12)
Turkmenistan	63 (31 to 102)	152 (54 to 270)	3.11(1.49 to 5.07)	3.58(1.27 to 6.34)	0.46(0.22 to 0.71)
Azerbaijan	238 (108 to 390)	543 (214 to 985)	4.40(1.99 to 7.22)	5.06(2.01 to 9.19)	0.48(0.23 to 0.73)
China	12,530 (6,219 to 19,835)	58,745 (24,601 to 99,889)	1.40(0.68 to 2.23)	2.81(1.20 to 4.76)	2.46(2.23 to 2.69)
Thailand	785 (418 to 1,226)	4,327 (1,873 to 7,192)	1.94(1.04 to 3.04)	3.99(1.74 to 6.59)	2.50(2.31 to 2.69)
Armenia	189 (75 to 311)	323 (127 to 542)	6.58(2.61 to 10.84)	7.33(2.91 to 12.24)	0.33(-0.02 to 0.68)
Sri Lanka	141 (65 to 222)	406 (156 to 738)	1.21(0.56 to 1.92)	1.50(0.58 to 2.72)	0.81(0.55 to 1.08)
Georgia	314 (140 to 506)	442 (176 to 737)	4.81(2.19 to 7.72)	7.44(3.00 to 12.34)	1.68(0.63 to 2.73)
Mauritius	20 (10 to 31)	80 (29 to 134)	2.50(1.19 to 3.9)	4.27(1.56 to 7.11)	2(0.98 to 3.02)
Kazakhstan	788 (352 to 1,259)	963 (401 to 1,580)	6.10(2.71 to 9.75)	5.39(2.23 to 8.82)	-0.46(-1.03 to 0.10)
Seychelles	2 (1 to 4)	8 (3 to 13)	4.18(1.68 to 6.81)	6.40(2.39 to 10.71)	1.38(1.00 to 1.77)
Malaysia	262 (112 to 409)	1,263 (479 to 2,064)	2.53(1.06 to 3.98)	4.40(1.67 to 7.15)	1.92(1.72 to 2.12)
Brunei Darussalam	4 (2 to 6)	20 (8 to 32)	3.38(1.49 to 5.35)	5.08(1.96 to 8.19)	1.41(1.28 to 1.54)
Singapore	50 (22 to 80)	241 (95 to 400)	2.04(0.90 to 3.28)	2.80(1.11 to 4.64)	1.10(0.76 to 1.44)
Taiwan (Province of China)	373 (162 to 594)	2,080 (801 to 3,503)	2.19(0.95 to 3.51)	4.93(1.90 to 8.28)	2.78(2.38 to 3.19)
Japan	4,513 (2,297 to 7,026)	10,265 (4,607 to 16,528)	2.51(1.28 to 3.90)	2.60(1.13 to 4.12)	0.08(-0.03 to 0.19)
Republic of Korea	768 (434 to 1,218)	2,558 (1,160 to 4,161)	2.42(1.38 to 3.81)	2.73(1.25 to 4.43)	0.40(0.20 to 0.59)

ASMR, age–standardized mortality rate; AAPC, average annual percentage change; UI, uncertainty intervals; CI, confidence intervals.

**Figure 2:**
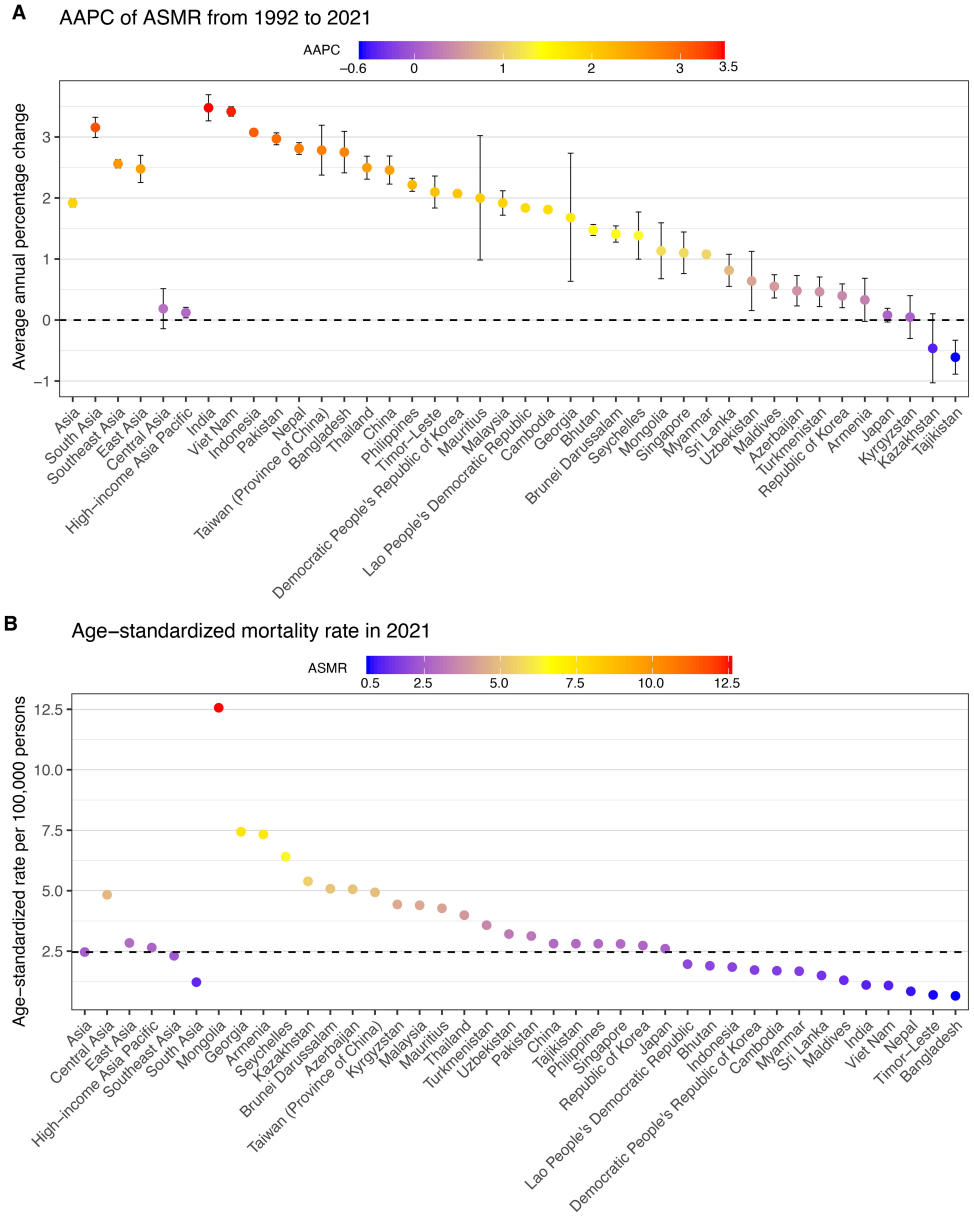
AAPC of mortality of total cancer attributable to high BMI across Asia, five GBD regions and 34 countries/territories from 1992 to 2021, with ASMR in 2021. **(A)** AAPC of ASMR from 1992 to 2021. **(B)** ASMR in 2021. AAPC, average annual percentage change; ASMR, age–standardized mortality rate.

From 1992 to 2021, cancer-related ASMR increased across four of the five GBD regions in Asia, while the trend in Central Asia remained relatively stable (**Table 1**, **Figures 1B**, **2A**). Among five GBD regions in Asia, East Asia, South Asia, and Central Asia exhibited a steadily increasing patterns in ASMR over time. The most substantial rise was observed in South Asia (AAPC: 3.16%, 95%: 2.99 to 3.32), although it maintained the lowest ASMR among all regions in 2021 (**Table 1**, **Figures 2A, B**). Southeast Asia also showed a notable increase (AAPC: 2.56%, 95%: 2.49 to 2.63), with ASMR more than doubling over the study period. East Asia reported the largest absolute number of deaths and a significant increase in ASMR (AAPC: 2.48%, 95%CI: 2.25 to 2.70) (**Table 1**, **Figures 1B**, **2A**). HIAP demonstrated the lowest AAPC (0.12%, 95%CI: 0.04 to 0.21) and a distinct temporal trajectory: its ASMR initially rose, peaked in 1997, and subsequently declined. (**Table 1**, **Figures 1B**, **2A**). In contrast, Central Asia demonstrated a fluctuating pattern with an initial increase until 2004, a decline thereafter, a rise again after 2007, and a further decline after 2011. Nonetheless, the overall trend remained stable (AAPC: 0.19%; 95% CI: –0.14 to 0.51) (**Table 1**, **Figures 1B**, **2A**).

**Figure 3:**
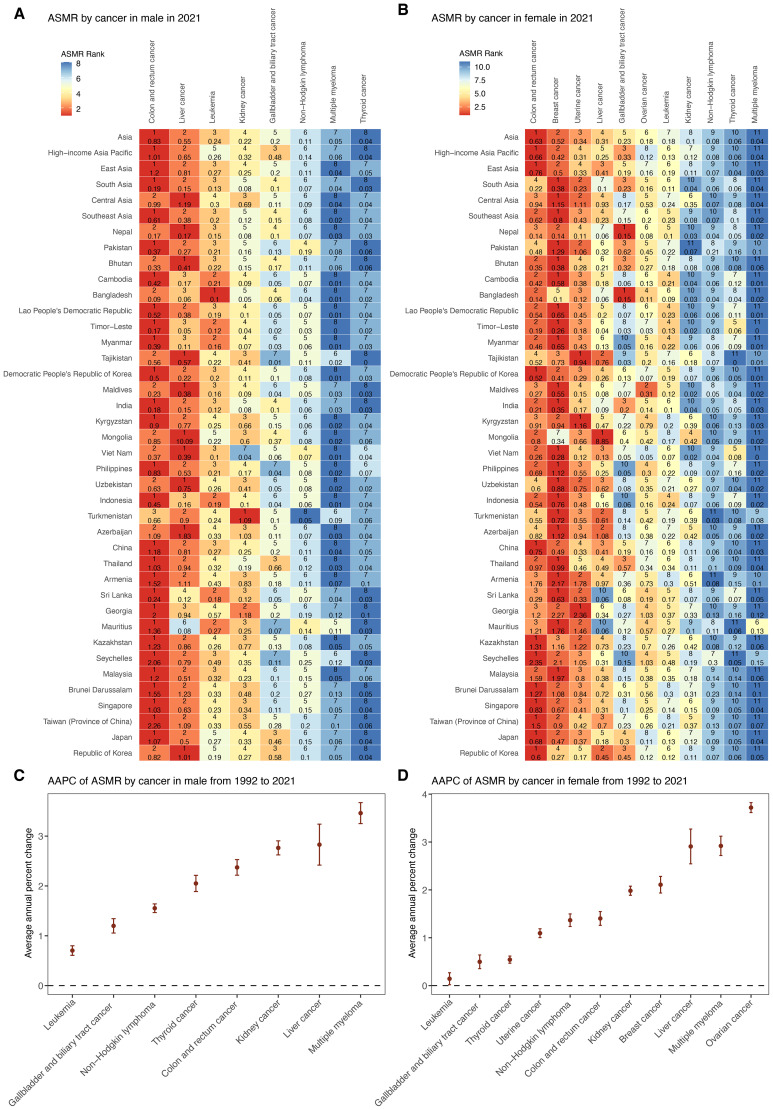
Trends in mortality of cancer subtypes attributable to high BMI by sex from 1992 to 2021. **(A)** ASMR of eight male cancer subtypes in 2021 among Asia, five GBD regions, and 34 countries and territories. **(B)** ASMR of 11 female cancer subtypes in 2021 among Asia, five GBD regions, and 34 countries and territories. **(C)** AAPC of ASMR for eight male cancer subtypes in Asia from 1992 to 2021. **(D)** AAPC of ASMR for 11 female cancer subtypes in Asia from 1992 to 2021. ASMR, age–standardized mortality rate; AAPC, average annual percentage change; APC, annual percentage change.

In 2021, cancer types attributable to high BMI presented varying ASMRs across sexes in Asia (**Figure 3**, **Supplementary Table S3**). Among males, colon and rectum cancer, liver cancer, and leukemia ranked as the leading causes of high-BMI-related cancer ASMR. Among females, colon and rectum cancer, breast cancer, and uterine cancer accounted for the highest ASMRs. Across both sexes, non-Hodgkin lymphoma, multiple myeloma and thyroid showed lowest ASMRs. Between 1992 and 2021, all cancer types showed increasing trends in ASMRs across both sexes (**Figure 3**, **Supplementary Table S3**). Among males, multiple myeloma demonstrated the highest AAPC (3.46%, 95% CI: 3.25 to 3.67) followed by liver cancer (2.83%, 95% CI: 2.42 to 3.24), and kidney cancer (2.76%, 95% CI: 2.62 to 2.91). For females, ovarian cancer had the fastest rise (3.72%, 95% CI: 3.62 to 3.82), followed by multiple myeloma (2.92%, 95% CI: 2.72 to 3.12) and liver cancer (2.91%, 95% CI: 2.54 to 3.27). In contrast, leukemia and gallbladder and biliary tract cancer exhibited the slowest ASMR increases in both sexes.

Across five GBD regions in Asia, ASMRs for major cancers showed substantial regional heterogeneity in 2021 (**Figure 3**, **Supplementary Table S4**). Central Asia recorded the highest ASMR for several cancers, including liver, uterine, and breast cancer, whereas South Asia consistently reported the lowest ASMR across most cancer types. In addition, the burden of male cancers such as colorectal and liver cancer, as well as female cancers such as breast and uterine cancer, was notably higher in HIAP and East Asia.

### 3.2 Decomposition analysis

**Figure 4:**
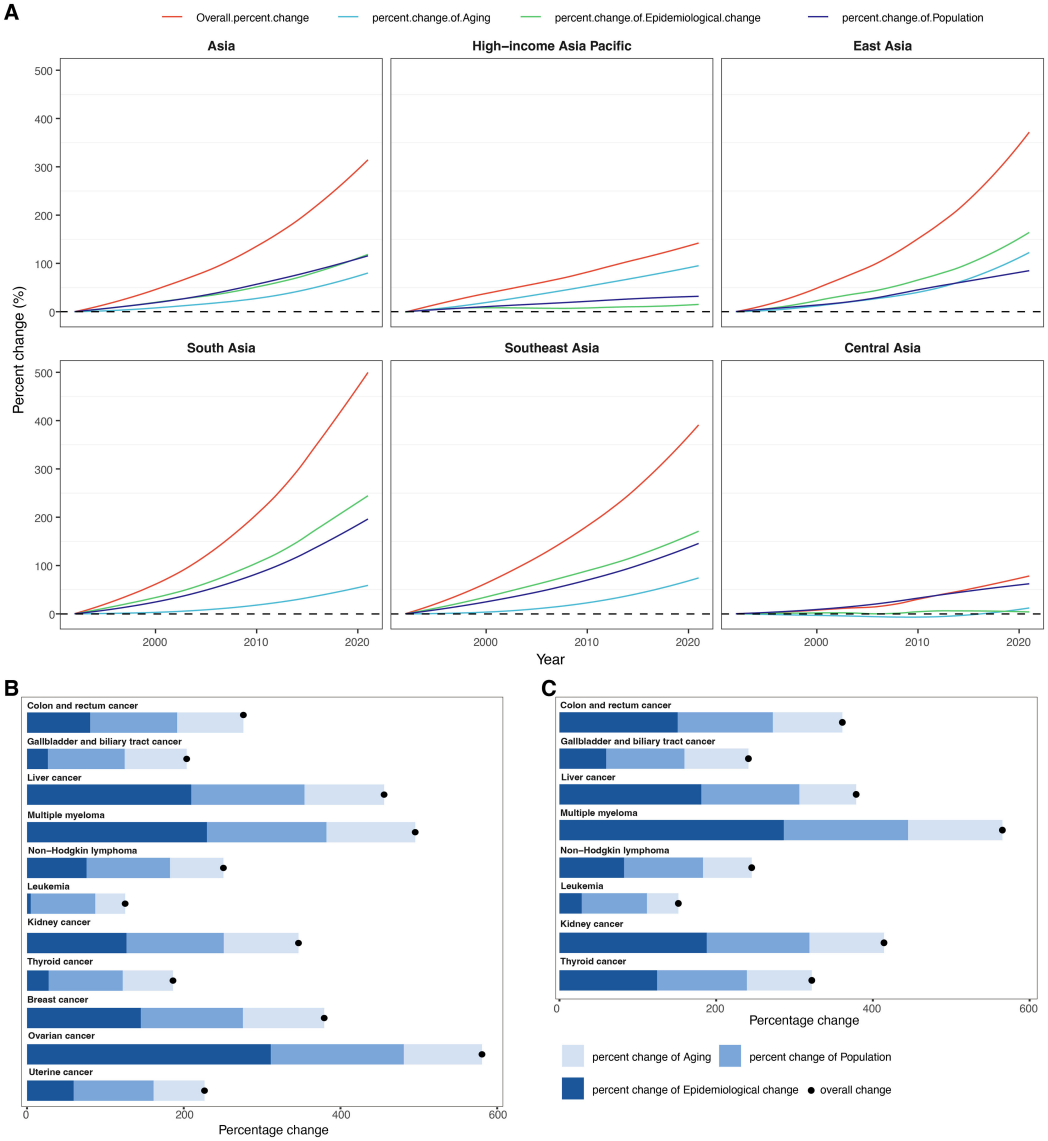
Results of decomposition analysis. **(A)** Decomposition of changes in cancer-related deaths attributable to high BMI from 1992 to 2021 within Asia and five GBD regions. The decomposition was conducted using the number of cancer-related deaths in 1992 as the reference for each year. **(B)** Decomposition of changes in deaths for 11 female cancer subtypes attributable to high BMI between 1992 and 2021 in Asia. **(C)** Decomposition of changes in deaths for eight male cancer subtypes attributable to high BMI between 1992 and 2021 in Asia.

From 1992 to 2021, the total deaths for cancers attributable to high BMI in Asia increased by 313.57% (**Figure 4A**, **Supplementary Table S5**). This substantial rise was primarily driven by epidemiological changes, contributing a 118.20% increase, followed closely by population growth (115.54%) and population aging (79.83%). Among the five GBD regions in Asia, South Asia recorded the highest overall percent increase in deaths at 493.26% between 1992 and 2021, driven primarily by the largest contributions from both epidemiological change (240.68%) and population growth (194.59%) among all regions, while Central Asia had the smallest overall increase (77.44%) and the lowest contributions from aging (11.83%) and epidemiological change (3.59%). Furthermore, East Asia experienced the largest aging-related increase (122.11%). In contrast, HIAP showed the lowest contribution from population growth (32.07%).

Across all cancer subtypes and both sexes, the most pronounced increase was observed for ovarian cancer in females, with an overall percent change of 580.59%, largely attributable to epidemiological changes (310.98%), followed by population growth (169.79%) and aging (99.81%) (**Figure 4B**, **Supplementary Table S6**). Similarly, in males, the highest percent change was noted for multiple myeloma, which rose by 565.26%, predominantly due to epidemiological change (286.20%), population growth (158.66%), and aging (120.41%) (**Figure 4C**, **Supplementary Table S6**). Conversely, leukemia exhibited the lowest overall percent change: 151.72% in males and 125.19% in females, primarily driven by demographic drivers such as population growth (83.28% in males; 82.34% in females) and aging (40.09% in males; 38.21% in females). Interestingly, thyroid cancer showed a steeper increase in males (322.03%) compared to females (186.22%), primarily due to a higher contribution of epidemiological change in males (124.50%) versus 27.46% in females. Similarly, for gallbladder and biliary tract cancer, the contribution of epidemiological change was more substantial in males (59.58%) than in females (26.57%). Overall, epidemiological changes contributed significantly to the increasing deaths burden, especially for multiple myeloma, ovarian cancer, and liver cancer. Conversely, cancers such as leukemia and thyroid cancer in females exhibited relatively low contributions from epidemiological change.

### 3.3 Trends in ASMR of total cancer across 34 Asian countries and territories

In 2021, the burden of total cancer mortality attributable to high BMI varied substantially across 34 Asian countries and territories (**Table 1**, **Figure 2**, **Supplementary Figures S1–3**). China reported the highest number of deaths, with an estimated 58,745 cases (95% UI: 24,601 to 99,889). Following China, India and Japan contributed significantly to the regional burden, with 13,180 deaths (95% UI: 5,945 to 20,947) and 10,265 deaths (95% UI: 4,607 to 16,528), respectively (**Table 1**, **Supplementary Figure S1**). However, the highest ASMRs were observed in Mongolia (12.56 per 100,000, 95% UI: 5.08 to 21.01), Georgia (7.44, 95% UI: 3.00 to 12.34), and Armenia (7.33, 95% UI: 2.91 to 12.24), despite lower absolute mortality counts (**Table 1**, **Figure 2B**, **Supplementary Figure S2**). In contrast, countries such as Bangladesh, Timor-Leste, and Nepal had the lowest ASDRs, with values of 0.65 (95% UI: 0.31 to 1.10), 0.69 (95% UI: 0.31 to 1.15), and 0.84 (95% UI: 0.41 to 1.39), respectively.

From 1992 to 2021, most countries exhibited increasing trends in ASMRs for high BMI-related cancers (**Table 1**, **Figure 2A**, **Supplementary Figure S3**). The largest AAPC was reported in India (3.48%, 95% CI: 3.27 to 3.69), Viet Nam (3.42%, 95% CI: 3.34 to 3.50), and Indonesia (3.07%, 95% CI: 3.03 to 3.12) (**Table 1**, **Figure 2A**, **Supplementary Figure S3**). Other nations with markedly rising trends included Bangladesh (2.75%, 95% CI: 2.41 to 3.09), Nepal (2.81%, 95% CI: 2.71 to 2.91), and Pakistan (2.97%, 95% CI: 2.87 to 3.07). Conversely, several countries experienced minimal or negative trends. Tajikistan (−0.61%, 95% CI: −0.89 to −0.33) showed decreasing trends, while Kazakhstan (−0.46%, 95% CI: −1.03 to 0.10), Kyrgyzstan (0.05%, 95% CI: −0.30 to 0.40), and Japan (0.08%, 95% CI: −0.03 to 0.19) displayed relatively stable rates over the period (**Table 1**, **Figure 2A**, **Supplementary Figure S3**).

### 3.4 Trends in mortality of total cancer across different age groups

**Figure 5:**
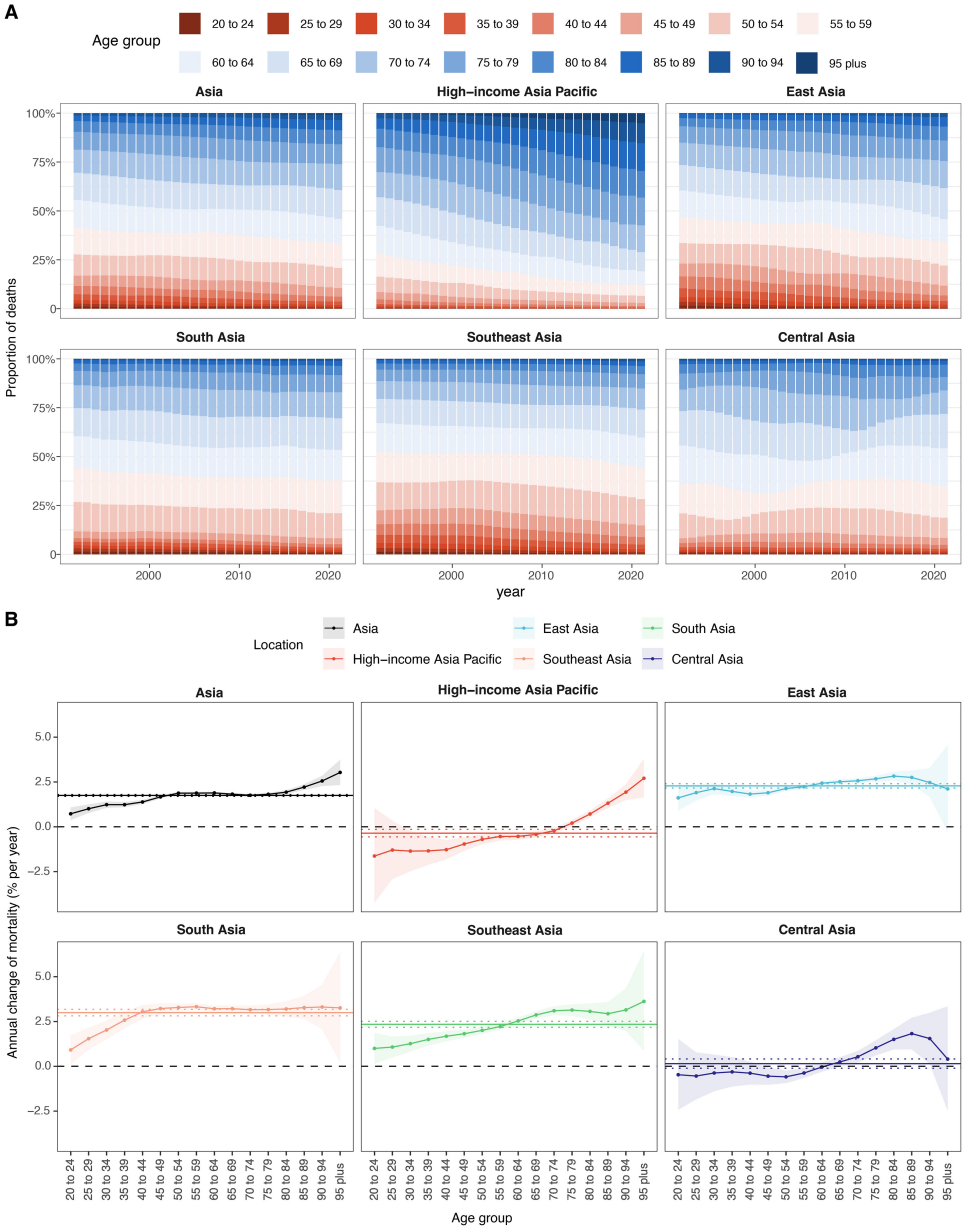
Local drifts of mortality rate and age distribution of cases within Asia and five GBD regions, 1992–2021. **(A)** Temporal change in the relative proportion of cancer deaths across 16 age groups, 1992–2021. **(B)** Local drifts of cancer mortality rate (estimates from age–period–cohort models) for 16 age groups, 1992–2021. The dots and shaded areas indicate the annual percentage change of mortality rate (% per year) and the corresponding 95% CIs.

From 1992 to 2021, the age distribution of BMI-attributable cancer deaths in Asia exhibited distinct temporal changes (**Figure 5A**, **Supplementary Table S7**). In 1992, the highest proportion was observed in the 60–64 age group (14.34%), followed by 55–59 (13.54%) and 65–69 (13.89%). By 2021, the 65–69 age group had the highest burden (14.72%), followed by 60–64 (12.55%) and 55–59 (12.58%). Across regions, the HIAP showed the largest increase in the 95+ age group, from 0.42% in 1992 to 5.15% in 2021. In East Asia, the peak proportion shifted from 13.46% in 55–59 (1992) to 15.74% in 65–69 (2021). In South Asia, the 55–59 group had the highest burden in both 1992 (17.42%) and 2021 (16.84%). In Southeast Asia, the maximum proportion was observed in 55–59 (15.57%) in 1992 and remained similar in 2021 (15.8%). Central Asia showed a consistently high burden in 60–64 years: 19.82% in 1992 and 19.57% in 2021. Among younger age groups (20–34 years), the proportions decreased across all regions. In Asia overall, the 20–24 group declined from 1.19% in 1992 to 0.35% in 2021. East Asia recorded a drop from 1.61% to 0.26%, and South Asia from 1.45% to 0.58%. HIAP maintained the lowest proportions across all years and age groups in this range. Among older age groups (≥75 years), proportions increased from 1992 to 2021 in most regions. In Asia, the 85–89 group rose from 2.89% to 5.21%, and the 90–94 group from 0.99% to 2.62%. In HIAP, the 85–89 group increased from 5.4% to 14.05%, and the 90–94 group from 2.19% to 10.35%.

From 1992 to 2021, the local drift of total cancer mortality attributable to high BMI in Asia showed an age-dependent increasing trend, rising from 0.73% per year (95% CI, 0.37 to 1.08) in the 20–24 age group to 3.03% (95% CI, 2.33 to 3.74) in individuals aged 95 years and above (**Figure 5B**, **Supplementary Table S8**). Among the subregions, South Asia recorded the highest drift values, exceeding 3.0% from ages 40 to 94 and peaking at 3.33% (95% CI, 3.14 to 3.51) at ages 55–59. Southeast Asia presented a similar upward pattern, with values ranging from 1.00% (95% CI, 0.14 to 1.87) at 20–24 to 3.62% (95% CI, 0.89 to 6.43) at ≥95. In East Asia, local drift increased steadily from 1.61% (95% CI, 0.91 to 2.32) at 20–24 to 2.83% (95% CI, 2.59 to 3.07) at 80–84, followed by 2.11% (95% CI, –0.26 to 4.54) at ≥95. In contrast, HIAP exhibited a distinctly different pattern, characterized by negative or flat local drifts in young and middle-aged adults. Specifically, mortality burden declined in age groups 20–69 years, with the steepest reductions observed at ages 20–24 (–1.63% per year; 95% CI, –4.23 to 1.04) and 30–34 (–1.36%; 95% CI, –2.47 to –0.23). However, positive local drifts emerged from age 75 onward, culminating in 2.71% (95% CI, 1.65 to 3.78) among individuals aged ≥95. Central Asia displayed a more complex and heterogeneous pattern. Local drift estimates were negative or near-zero for individuals aged 20–64 years, with the lowest value at –0.59% (95% CI, –0.93 to –0.24) for those aged 50–54. Positive drifts appeared only in individuals aged ≥70 years, peaking at 1.83% (95% CI, 0.95 to 2.71) among the 85–89 age group.

### 3.5 Age, period, and birth cohort effects on the ASMR of total cancer

#### 3.5.1 Age effect

**Figure 6:**
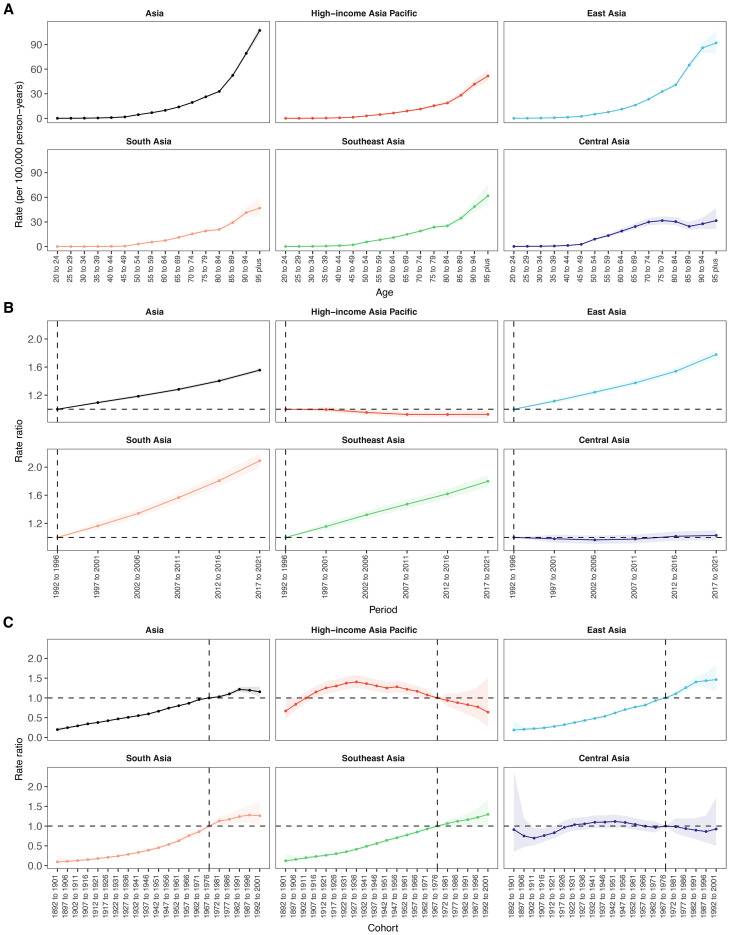
Age, period and birth cohort effects on mortality attributable to high BMI within Asia and five GBD regions by age–period–cohort models. **(A)** Age effects are depicted by the fitted longitudinal age curves of incidence rate (per 100–000 person-years) adjusted for period deviations. **(B)** Period effects are demonstrated by the relative risk of mortality rate (mortality rate ratio) and computed as the ratio of age-specific rates from 1992–1996 to 2017–2021, with the referent cohort set at 1992–1996. **(C)** Birth cohort effects are shown by the relative risk of mortality rate and computed as the ratio of age-specific rates from the 1892–1902 cohort to the 1992–2001 cohort, with the referent cohort set at 1892–1902. The dots and shaded areas denote incidence rates or rate ratios and their corresponding 95% CIs.

Age-specific mortality rates of total cancer attributable to high BMI increased steadily with age across Asia and all five GBD subregions (**Figure 6A**, **Supplementary Table S9**). In Asia overall, the rate rose from 0.10 per 100,000 in the 20–24 age group to 107.14 in the 95+ group. East Asia had the highest rates at all ages, peaking at 91.98 in those aged 95+, while South Asia had the lowest (46.84 in 95+). Southeast Asia showed moderate rates (from 0.10 to 61.82). Central Asia displayed relatively high early-age rates (0.19 in 20–24) but a slower increase in older age (31.61 in 95+). HIAP had the lowest late-life burden among all subregions (51.60 in 95+), with a narrower age-related gradient.

#### 3.5.2 Period effect

From 1992 to 2021, the period effect on BMI-attributable total cancer mortality in Asia increased steadily, with the rate ratio rising from 1.00 (reference, 1992–1996) to 1.56 (95% CI: 1.53 to 1.58) during 2017–2021 (**Figure 6B**, **Supplementary Table S10**). Among the subregions, South Asia exhibited the most pronounced upward trend, with the rate ratio more than doubling from 1.00 in 1992–1996 to 2.09 (95% CI: 1.99 to 2.19) by 2017–2021. East Asia followed a similar pattern, with the period effect increasing from 1.00 to 1.78 (95% CI: 1.72 to 1.84), while Southeast Asia showed a rise to 1.80 (95% CI: 1.72 to 1.88) in the final period. In contrast, HIAP showed a declining trend, with the period effect decreasing from 1.00 in the reference period to 0.93 (95% CI: 0.88 to 0.98) in 2017–2021. Similarly, Central Asia remained relatively stable throughout, fluctuating near 1.00 and reaching 1.03 (95% CI: 0.96 to 1.10) in the most recent period.

#### 3.5.3 Cohort effect

Across Asia, cohort effects on cancer mortality attributable to high BMI showed a consistent upward trend across successive birth cohorts, with rate ratios rising from 0.20 (95% CI: 0.16 to 0.25) in the 1892–1901 cohort to a peak of 1.22 (95% CI: 1.16 to 1.28) in the 1982–1991 cohort, followed by a slight decline (**Figure 6C**, **Supplementary Table S11**). East, South, and Southeast Asia exhibited similar upward trends, with cohort risks exceeding 1.00 after 1967–1976. In East Asia, rate ratios increased from 0.19 (95% CI: 0.08 to 0.42) in the earliest cohort to 1.46 (95% CI: 1.18 to 1.82) in the 1992–2001 cohort, showing the highest cohort-related risk increase among all regions. Southeast Asia exhibited a comparable trajectory, with the rate ratio increasing from 0.12 (95% CI: 0.05 to 0.30) in the 1892–1901 cohort to 1.30 (95% CI: 1.00 to 1.69) in the most recent cohort. South Asia presented the lowest starting risk but demonstrated the steepest proportional rise across cohorts, from 0.10 (95% CI: 0.03 to 0.26) to 1.26 (95% CI: 1.00 to 1.60). In contrast, an inverted U-shaped pattern was observed in HIAP, where the cohort effect peaked earlier, reaching 1.41 (95% CI: 1.25 to 1.58) in the 1927–1936 cohort and gradually declined to 0.64 (95% CI: 0.27 to 1.50) in the 1992–2001 cohort. Similarly, Central Asia demonstrated a modest peak in the 1942–1951 cohort (1.12; 95% CI: 0.97 to 1.29), followed by a decline to 0.93 (95% CI: 0.50 to 1.72) in the 1992–2001 cohort.

## 4 Discussion

Applying an age-period-cohort model, our investigation presents the first comprehensive evaluation of long-term trends in cancer mortality attributable to high BMI specifically in Asia. Over the 30-year period, we observed an almost fourfold increase in annual cancer deaths and a near doubling of age-standardized mortality rates. Significant heterogeneity was noted across subregions and population groups. South Asia exhibited the most rapid increase in ASMR, despite low absolute rates, while the HIAP region experienced an initial rise, peaking in the late 1990s, and then a decline. Among cancer sites, high BMI-related colon and rectal cancers dominated mortality in both sexes, along with liver and leukemia in men and breast and uterine cancers in women. Other cancers linked to high BMI, such as ovarian cancer in women and multiple myeloma in men, also showed large relative increases. The age-period-cohort analysis highlights steep increases in risk with age, especially in older adults. Successive birth cohorts born more recently have markedly higher mortality risk than earlier generations. For example, generations born after 1960 generally faced higher risks, particularly in East, South, and Southeast Asia. Conversely, the HIAP region shows an inverted U-shaped cohort trend, with the highest risk in mid-20th century cohorts and a subsequent decline in younger cohorts. Period effects rose steadily in most regions, except in HIAP where recent period risks declined. These findings reveal a significant increase in high BMI-related cancer mortality in Asia, with notable variations observed across different regions, sexes, and cancer types.

The substantial rise in high BMI-attributable cancer mortality in Asia parallels a rapid “nutrition transition” across the region. Economic development, urbanization, and globalization have driven profound lifestyle changes, such as diets rich in sugar, fats, and processed foods, along with increasingly sedentary behaviors, which have fueled the obesity epidemic in Asia (28). Worldwide, the obesity crisis is well documented. The World Health Organization (WHO) reports that global adult obesity has more than doubled since 1990 (29), and the World Obesity Federation even projects a rise of obesity prevalence from 14% to 24% of the population between 2020 and 2035, affecting nearly 2 billion people by 2035 (30). Previous global studies found only modest increases in age-standardized cancer mortality from high BMI. For example, global age-adjusted rates of obesity-attributable cancers grew by only 0.48% per year from 2010 to 2019 (5). In contrast, we observed near 2% annual increases in Asia’s ASMR. This region-specific increase in high BMI-attributable cancer mortality likely reflects the rapid adoption of Western lifestyle patterns in Asia. Consistent with this, Westernized diets and processed foods now characterize large parts of Asia. For instance, after industrialization East Asia reached an estimated 40.9% adult prevalence of overweight or obesity (28). The obesity epidemic is, therefore, a key underlying driver of the rising cancer death trends, alongside improved cancer detection and reporting. Our findings that high BMI-related cancer mortality climbed much more steeply in Asia than global averages align with previous GBD analyses noting the rapid growth of high-BMI disease burden in Asia and the urgent need for targeted interventions (31, 32).

Marked differences emerged across Asian subregions. South Asia experienced the fastest increases in obesity-linked cancer mortality, though it still had the lowest absolute ASMR in 2021. This pattern suggests South Asia is in an earlier but rapidly accelerating stage of the high BMI related cancer transition. Southeast Asia likewise saw a notable upward trend. Rapid urbanization and dietary change, such as shifts from traditional whole-grain diets toward highly processed, high-calorie foods, have driven obesity and diabetes upward in South Asia and Southeast Asia (33). Indeed, WHO notes that South-East Asia historically had some of the lowest obesity rates globally, but these rates are now rising alarmingly in the last decade (34). In practical terms, South Asia’s large population has started to accumulate weight-related cancer risk, with demographic expansion compounding the effect. East Asia bears the largest absolute burden, with China alone accounting for approximately 48% of deaths in 2021, and its ASMR has also climbed significantly. By contrast, HIAP demonstrated a unique trend, characterized by a rise in ASMR until the late 1990s, followed by a decline. The plateau-and-decline pattern is consistent with trends in many high-income countries, where obesity prevalence has stabilized or decreased due to public health initiatives and a cultural emphasis on healthy weight. For example, since 2008, Japan has operated a nationwide Specific Health Check-ups program that requires all adults aged 40–74 to undergo annual waist-circumference measurement and receive counselling if at risk of metabolic syndrome. Participation is mandatory regardless of insurance type, and the costs are fully covered by insurers (35, 36). This policy exemplifies a population-wide screening and lifestyle-modification approach to reduce obesity and its downstream health consequences. In December 2022, Singapore introduced a color−coded Nutri−Grade labelling system for pre−packaged beverages. The labels grade drinks from A to D based on sugar and saturated−fat content and are intended to help consumers choose lower−sugar options. Furthermore, WHO and experts recommend evidence-based strategies, such as restricting the marketing of unhealthy foods and promoting urban planning that encourages walking and exercise (37).

Globally, colorectal, breast, liver, and uterine are among the top high BMI-related cancers, and our Asian results reflect that pattern (5). In men, colorectal cancer accounted for the largest share of high BMI-attributable deaths, followed by liver cancer and, unexpectedly, leukemia. In women, colorectal, breast, and uterine cancers were the leading contributors. These findings align well with global evidence that excess adiposity is a well-established risk factor cancer (2), and that obesity-driven insulin resistance and inflammation can promote carcinogenesis. The prominence of liver cancer in obese men likely reflects the combined effects of metabolic dysfunction and preexisting liver disease, as obesity could exacerbate nonalcoholic fatty liver disease and steatohepatitis, which are known precursors of hepatocellular carcinoma. In women, the strong impact of high BMI on postmenopausal breast and uterine cancer is expected, as fat tissue produces extra estrogen and inflammation that fuel these hormone-sensitive tumors. For example, higher adiposity has been shown to raise estrogen levels markedly, significantly increasing breast and uterine cancer risk (38–40). One Asia-Pacific Cohort Study similarly found that overweight Asians faced significantly higher mortality from colon, rectum, breast, and other cancers (41). Overall, every high BMI-related cancer type we studied showed increasing ASMR trends in both sexes, underscoring that the obesity pandemic is broadening the cancer burden across many tumor sites.

Although most of our findings were consistent with global evidence, the results for liver cancer diverge from those reported by the Asia Pacific Cohort Studies Collaboration (APCSC). Drawing on cohorts established between the 1970s and 1990s with a median follow-up of only four years, the APCSC reported essentially no association between BMI and liver cancer mortality after adjustment for age, sex, smoking, and alcohol use (42). By contrast, our analysis, based on the GBD 2021 dataset, indicated a substantial rise in ASMR of liver cancer due to high BMI between 1992 and 2021. Several factors may explain this discrepancy. First, APCSC participants were relatively lean and were followed for a median of only four years. Thus, the cohorts largely pre-date the modern obesity epidemic and may not capture the long latency between metabolic dysfunction and hepatocarcinogenesis. Second, APCSC lacked data on hepatitis B and C infections, which are highly prevalent in Asia and may interact with obesity to accelerate hepatocarcinogenesis, potentially leading to regional heterogeneity. Third, our estimates derive from the GBD model, which integrates contemporary data on high-BMI prevalence and relative risks from multiple cohorts and meta-analyses. This approach reflects the rising burden of non-alcoholic fatty liver disease, steatohepatitis and diabetes, factors that have become increasingly important drivers of hepatocellular carcinoma over the past three decades. Furthermore, our findings indicate that liver cancer mortality attributable to high BMI in Asia is increasing, in line with recent meta-analyses showing that high BMI significantly increases the risk of primary liver cancer incidence and mortality (43). Further cohort studies with contemporary data, detailed risk factor assessment, and longer follow-up are warranted to better elucidate these complex associations and to inform prevention strategies targeting metabolic liver disease.

Our decomposition analysis showed that the dominant driver of the rise in high−BMI–related cancer deaths in Asia was epidemiological change, which contributed 118 % to the net increase, whereas population growth and population ageing contributed 115 % and 80 %, respectively. This finding implies that exposure to high BMI, rather than demographic expansion, was chiefly responsible for the observed increase in deaths, highlighting that most of these deaths are preventable. Our results align with comparable decomposition studies but also exhibit differences. For instance, a global decomposition of colorectal cancer attributable to high BMI found that population growth was the primary driver of increasing burden. In high-SDI regions such as Western Europe and high-income North America, epidemiological change was found to reduce the burden of colorectal cancer attributable to high BMI, likely reflecting successful risk reduction through public health interventions and better weight control (44). Globally, population growth is the main driver of alcohol- attributable liver cancer, whereas epidemiological changes are the primary force behind high-BMI-related liver cancer (45). For tobacco-attributable lung cancer, population growth was again the chief driver of global deaths and DALYs, while epidemiological improvements reduced the burden in high−SDI regions (46). Collectively, these comparisons highlight that while demographic factors often dominate in many contexts, our finding that epidemiological change is the principal driver across multiple high-BMI–related cancers in Asia underscores the critical role of obesity prevention and lifestyle interventions. This echo conclusions from other Asian analyses that call for concerted action on obesity. For example, Chen et al. emphasized the urgent need for governments and societies to “enhance awareness” of high BMI risks and reduce its impact in Asia (31).

The age-period-cohort analysis provides further insights into who is most at risk and why. As expected, the age effect shows a steep rise in mortality with advancing age across all GBD regions. In 2021 Asia-wide, ASMR were near zero among young adults but rose sharply in older ages, with rates among those aged 95 and older being two to three orders of magnitude higher. This is consistent with cancer being largely a disease of aging (47). The period effect rose steadily across East, South and Southeast Asia, likely driven by worsening high-BMI prevalence and other risk factors. However, the period effect declined in HIAP in recent years, suggesting that broad improvements such as stabilized obesity rates, effective prevention campaigns, or better treatments have lowered the overall death risk in each calendar period. Indeed, our results show that HIAP had the lowest AAPC and followed a distinct trajectory: its ASMR increased in the early 1990s, peaked in 1997, and subsequently declined. We propose a “trajectory lag” hypothesis: HIAP’s decline may represent a future trajectory for other Asian regions as they progress through the obesity transition. The cohort effects support this view. For most regions, individuals born in the 1980s–1990s face higher mortality at a given age than those born earlier, reflecting rising childhood and adolescent obesity and sedentary lifestyles. East, South and Southeast Asia already show cohort rate ratios exceeding 1·0. In HIAP, however, the cohort effect exhibits an inverse-U pattern, with risk peaking among those born around the 1930s and declining in more recent cohorts, consistent with early public-health gains and a potential ceiling effect once the population achieved leanness. Local drift analyses reinforce these patterns: most Asian subregions show positive drift across older ages, whereas HIAP has declining mortality trends in young and middle-aged adults. Together, these findings suggest that other Asian regions may follow HIAP’s trajectory but with a time lag, highlighting an urgent window for pre-emptive policy action. By implementing evidence-based obesity prevention, promoting healthy lifestyles, and improving access to screening and treatment, policymakers could replicate HIAP’s successes and avert a future surge in high-BMI-related cancer mortality.

These results clearly point to an urgent need for targeted prevention and control strategies to curb the high BMI-driven cancer epidemic. However, given Asia’s profound heterogeneity in cultural norms, economic development, and health system readiness, a one-size-fits-all approach is unlikely to succeed. For instance, obesity rates are relatively stable in HIAP, but the population is aging. Efforts could focus on strengthening health systems and developing more sports facilities and parks to provide convenient exercise options for older adults (48, 49). In East Asia, rapid industrialization has caused severe air pollution and altered traditional diets (50, 51). Policies should concentrate on enhancing air pollution control and improving air quality, while promoting outdoor physical activity. A diet low in fat and sugar but high in fiber should be encouraged, as well as mental health education to mitigate stress-related obesity (28). In Southeast Asia, where the informal food sector is dominant, regulating the advertising and marketing of unhealthy foods, providing nutrition information, and implementing front-of-package labels may be most effective in promoting healthier food choices. Moreover, in some low-income Asian countries, educational courses, posters, and brochures could be used to raise public awareness of the link between obesity and cancer, reduce the stigma associated with cancer diagnoses, and enhance early detection of precancerous lesions (52, 53). Training community health workers in weight management and health counseling might increase the coverage of obesity and cancer control programs. National efforts to expand cancer screening and health insurance coverage can ease the financial burden on cancer patients (52, 54). At the same time, cancer control programs should adapt to this evolving profile. Enhanced screening could mitigate the impact of weight-associated cancers. Expanding colorectal cancer screening in populations with rising obesity rates and instituting surveillance for non-alcoholic fatty liver disease in obese patients can enable early detection of both colorectal and liver cancers. Clinical guidelines should emphasize weight management as part of cancer prevention and survivorship. Furthermore, encouraging healthy weight before and after pregnancy to reduce future breast and endometrial cancer risk in women. For men, addressing obesity alongside other risk factors, including hepatitis immunization, and reducing alcohol and tobacco use, could help prevent colorectal and liver cancers. Additionally, our cohort study reveals that younger generations are at a disproportionately higher risk, underscoring the importance of establishing healthy habits early. Promising interventions include school- and university-based programs that promote healthy eating and physical activity, as well as mobile digital health apps for monitoring physical activity in adolescents. Fiscal policies, such as subsidies for fruits and taxes on ultra-processed foods, may further improve dietary habits in this cohort. Such coordinated, culturally tailored, and multi-faceted strategies, in line with WHO’s Global Action Plan for the Prevention and Control of Noncommunicable Diseases, are needed to mitigate the “silent pandemic” of high BMI-related cancers in Asia (53).

### 4.1 Limitations

Despite using the most up-to-date estimates from the GBD 2021 study, our analysis still has several limitations. First, the study is based on modeled estimates derived from heterogeneous sources, including cancer registries, household surveys, and cause-of-death statistics. In many low- and middle-income countries across Asia, cancer surveillance remains sparse or incomplete, and vital registration systems are underdeveloped. This data paucity increases reliance on statistical modeling, which may introduce bias or widen uncertainty, particularly in countries with little or no direct data input. The resulting estimates should be interpreted with caution, as reflected in the wide 95% uncertainty intervals around many outcomes. Second, the use of population-level epidemiological data to estimate attributable deaths carries an inherent risk of ecological fallacy. The comparative risk assessment framework employed by GBD attributes cancer mortality to high BMI based on average exposure levels and relative risks but cannot establish causality at the individual level. Residual confounding from factors such as diet quality, alcohol use, physical activity, or socioeconomic status may distort true associations. Third, secular improvements in cancer detection, staging, and treatment over the past three decades may have modified mortality outcomes independently of changes in high BMI exposure. These shifts may partly account for the favorable period effects observed in high-income settings and should be acknowledged as potential confounders. Fourth, we focused on high BMI as the main risk factor. However, obesity often co-occurs with other risk factors, such as poor diet, physical inactivity, and metabolic syndrome, and we did not account for these interactions in our analysis. Finally, our data end in 2021 and thus only partly capture the COVID-19 period. Pandemic-related lockdowns may have increased sedentary behavior and weight gain in 2020–2021. If these effects persist, future data may show a larger high BMI-related cancer burden.

## 5 Conclusion

In conclusion, over the past three decades Asia has experienced a dramatic rise in cancer mortality attributable to high BMI, driven largely epidemiological change rather than just population growth or ageing. The substantial heterogeneity observed across countries, sexes, and cancer types highlights the need for targeted strategies. Importantly, the trajectory observed in HIAP suggests that other regions may still be in earlier phases of a similar path. This “trajectory lag” offers a critical window for timely, preventive action. Given that obesity is a modifiable risk factor, its management through improved diet and increased physical activity is essential. Furthermore, incorporating BMI reduction targets into national cancer control plans and enhancing public health campaigns on the cancer risks of high BMI are also necessary to avert further increases and achieve long-term reductions in cancer burden.

## Data Availability

The original contributions presented in the study are included in the article/**Supplementary Material**. Further inquiries can be directed to the corresponding author.
