# Supplementary material for: Temporal trends in cancer mortality attributable to high BMI in Asia: an age-period-cohort analysis based on GBD 1992–2021
Source: Front Endocrinol (Lausanne). 2025 Oct 10;16:1691487. doi: 10.3389/fendo.2025.1691487 (PMC12549300; doi:10.3389/fendo.2025.1691487)

Temporal trends in cancer mortality attributable to high BMI in Asia: an age–period–cohort analysis based on GBD 1992–2021

Minguang Huang[1], Liejiong Wang[1], Ying Lou[2], Zhaoqi Qiu[1], Shengjian Yu[1], Feng Xuan[1,*]

[1]Department of Radiation Oncology, Zhuji Affiliated Hospital of Wenzhou Medical University, Shaoxing, China

[2]Department of Medical Oncology, Zhuji Affiliated Hospital of Wenzhou Medical University, Shaoxing, China

*** Correspondence:** Feng Xuan

Email: xfeng8901@outlook.com

Table S1. The International Classification of Diseases (ICD) codes for 11 cancer subtypes in the Global Burden of Disease (GBD) 2021 study. 3

Table S2. Classification of 34 countries and territories into 5 Asian Global Burden of Disease (GBD) regions in the GBD 2021 study. 4

Table S3. Trends in mortality of 11 cancer subtypes attributable to high BMI by sex in Asia from 1992 to 2021. 5

Table S4. Number and age–standardized rate of mortality for 11 cancer subtypes attributable to high BMI by sex in five GBD regions in Asia in 2021. 6

Table S5. The changes in deathes of total cancer attributable to high BMI associated with population ageing, population growth, and epidemiological change between 1992 and 2021 in Asia, five GBD regions, and 34 countries and territories. 11

Table S6. The changes in deathes of 11 cancer subtypes attributable to high BMI associated with population ageing, population growth, and epidemiological change between 1992 and 2021 in Asia. 12

Table S7. Proportion of deaths for total cancer attributable to high BMI across 16 age groups in 1992 and 2021 within Asia and five GBD regions. 14

Table S8. The local drift of mortality from 1992 to 2021 for total cancer attributable to high BMI across 16 age groups within Asia and five GBD regions. 16

Table S9 Age effects on mortality of total cancer attributable to high BMI across Asia and five GBD regions. 21

Table S10. Period effects on mortality of total cancer attributable to high BMI across Asia and five GBD regions. 26

Table S11. Cohort effects on mortality of total cancer attributable to high BMI across Asia and five GBD regions. 28

Figure S1. Deaths of total cancer attributable to high BMI in 2021 across 34 Asian countries and territories. 34

Figure S2. ASMR of total cancer attributable to high BMI in 2021 across 34 Asian countries and territories. 35

Figure S3. AAPC of ASMR for total cancer attributable to high BMI from 1992 to 2021 across 34 Asian countries and territories. 36

Table S1. The International Classification of Diseases (ICD) codes for 11 cancer subtypes in the Global Burden of Disease (GBD) 2021 study.

Cancers	ICD10	ICD9
Breast cancer	C50-C50.9, D05-D05.9, D24-D24.9, D48.6, D49.3	174-175.9, 217-217.8, 233.0, 238.3, 239.3, 610-610.9
Colon and rectum cancer	C18-C21.9, D01.0-D01.3, D12-D12.9, D37.3-D37.5	153-154.9, 209.1, 209.5, 211.3-211.4, 230.3-230.6, 569.0
Gallbladder and biliary tract cancer	C23-C24.9, D13.5	156-156.9
Kidney cancer	C64-C65.9, D30.0-D30.1, D41.0-D41.1	189.0-189.1, 189.5-189.6, 223.0-223.1
Leukemia	C91-C93.7, C93.9-C95.2, C95.7-C95.92	204-208.92, V10.59-V10.69, V16.6
Liver cancer	C22-C22.8, D13.4	155-155.1, 155.3-155.9, 211.5
Multiple myeloma	C88-C90.32	203-203.9
Non-Hodgkin lymphoma	C82-C85.29, C85.7-C86.6, C96-C96.9	200-200.9, 202-202.98
Ovarian cancer	C56-C56.9, D27-D27.9, D39.1	183-183.0, 220-220.9, 236.2
Thyroid cancer	C73-C73.9, D09.3, D09.8, D34-D34.9, D44.0	193-193.9, 226-226.9
Uterine cancer	C54-C54.9, D07.0-D07.2, D26.1-D26.9	182-182.9, 233.2

List of International Classification of Diseases (ICD) codes mapped to the Global Burden of Diseases cause list for causes of death.

Table S2. Classification of 34 countries and territories into 5 Asian Global Burden of Disease (GBD) regions in the GBD 2021 study.

Five GBD regions	34 countries and territories
High - income Asia Pacific	Republic of Korea; Japan; Singapore; Brunei Darussalam.
East Asia	China; Taiwan (Province of China); Democratic People's Republic of Korea.
Southeast Asia	Malaysia; Seychelles; Mauritius; Thailand; Indonesia; Uzbekistan; Philippines; Viet Nam; Myanmar; Timor - Leste; Lao People's Democratic Republic; Cambodia; Maldives.
Central Asia	Kazakhstan; Georgia; Armenia; Azerbaijan; Turkmenistan; Mongolia; Kyrgyzstan; Tajikistan.
South Asia	India; Bangladesh; Bhutan; Pakistan; Nepal.

Table S3. Trends in mortality of 11 cancer subtypes attributable to high BMI by sex in Asia from 1992 to 2021.

Sex	Cause	Number of mortality (95%UI)		ASMR (per 100,000 population,95%UI)		Trend
		In 1992	In 2021	In 1992	In 2021	AAPC (95%CI, %)
Male	Colon and rectum cancer	4,119 (1,413 to 6,757)	18,989 (7,613 to 31,216)	0.42(0.14 to 0.69)	0.83(0.33 to 1.36)	2.37(2.22 to 2.53)
Male	Gallbladder and biliary tract cancer	1,296 (870 to 1,879)	4,423 (2,577 to 6,423)	0.14(0.09 to 0.2)	0.2(0.12 to 0.29)	1.2(1.05 to 1.34)
Male	Liver cancer	2,854 (1,239 to 4,612)	13,661 (5,417 to 23,880)	0.24(0.11 to 0.4)	0.55(0.22 to 0.95)	2.83(2.42 to 3.24)
Male	Multiple myeloma	159 (-37 to 403)	1,058 (-298 to 2,793)	0.02(0 to 0.04)	0.05(-0.01 to 0.12)	3.46(3.25 to 3.67)
Male	Non-Hodgkin lymphoma	734 (269 to 1,212)	2,534 (865 to 4,391)	0.07(0.03 to 0.11)	0.11(0.04 to 0.19)	1.55(1.47 to 1.64)
Male	Leukemia	2,232 (1,472 to 3,161)	5,617 (3,850 to 7,926)	0.19(0.13 to 0.28)	0.24(0.16 to 0.33)	0.7(0.61 to 0.8)
Male	Kidney cancer	984 (384 to 1,601)	5,061 (1,971 to 8,450)	0.1(0.04 to 0.16)	0.22(0.09 to 0.37)	2.76(2.62 to 2.91)
Male	Thyroid cancer	240 (168 to 334)	1,013 (685 to 1,369)	0.02(0.02 to 0.03)	0.04(0.03 to 0.06)	2.05(1.89 to 2.21)
Female	Breast cancer	2,993 (-92 to 6,219)	14,344 (-497 to 28,793)	0.29(-0.01 to 0.59)	0.52(-0.02 to 1.05)	2.11(1.93 to 2.28)
Female	Ovarian cancer	726 (4 to 1,578)	4,939 (880 to 9,397)	0.06(0 to 0.14)	0.18(0.03 to 0.35)	3.72(3.62 to 3.82)
Female	Uterine cancer	2,784 (1,871 to 3,853)	9,089 (6,036 to 12,713)	0.25(0.17 to 0.34)	0.34(0.23 to 0.48)	1.1(1 to 1.19)
Female	Colon and rectum cancer	4,398 (1,661 to 7,186)	16,541 (7,132 to 27,118)	0.43(0.16 to 0.7)	0.63(0.27 to 1.04)	1.4(1.26 to 1.55)
Female	Gallbladder and biliary tract cancer	1,958 (1,324 to 2,793)	5,946 (3,634 to 8,579)	0.2(0.13 to 0.28)	0.23(0.14 to 0.33)	0.5(0.35 to 0.64)
Female	Liver cancer	1,475 (615 to 2,415)	8,198 (3,403 to 13,887)	0.14(0.06 to 0.22)	0.31(0.13 to 0.52)	2.91(2.54 to 3.27)
Female	Multiple myeloma	171 (-52 to 469)	1,017 (-349 to 2,565)	0.02(-0.01 to 0.04)	0.04(-0.01 to 0.1)	2.92(2.72 to 3.12)
Female	Non-Hodgkin lymphoma	573 (207 to 989)	2,009 (682 to 3,508)	0.05(0.02 to 0.09)	0.08(0.03 to 0.13)	1.37(1.23 to 1.5)
Female	Leukemia	2,114 (1,496 to 2,982)	4,759 (3,217 to 6,576)	0.18(0.13 to 0.25)	0.18(0.12 to 0.25)	0.14(0.02 to 0.27)
Female	Kidney cancer	612 (233 to 994)	2,732 (1,074 to 4,581)	0.06(0.02 to 0.1)	0.1(0.04 to 0.18)	1.98(1.89 to 2.08)
Female	Thyroid cancer	555 (398 to 750)	1,588 (1,103 to 2,159)	0.05(0.04 to 0.07)	0.06(0.04 to 0.08)	0.54(0.47 to 0.62)

Table S4. Number and age–standardized rate of mortality for 11 cancer subtypes attributable to high BMI by sex in five GBD regions in Asia in 2021.

Sex	Location	Cause	Number of mortality (95%UI)		ASMR (per 100,000 population,95%UI)	
			In 1992	In 2021	In 1992	In 2021
Male	High-income Asia Pacific	Colon and rectum cancer	786 (277 to 1,279)	2,113 (797 to 3,400)	0.87(0.3 to 1.42)	1.01(0.38 to 1.62)
Male	East Asia	Colon and rectum cancer	2,123 (674 to 3,554)	11,682 (4,548 to 20,226)	0.51(0.16 to 0.85)	1.2(0.47 to 2.05)
Male	South Asia	Colon and rectum cancer	235 (77 to 383)	1,345 (522 to 2,120)	0.07(0.02 to 0.12)	0.19(0.07 to 0.29)
Male	Central Asia	Colon and rectum cancer	185 (75 to 302)	348 (147 to 559)	0.92(0.38 to 1.49)	0.99(0.42 to 1.6)
Male	Southeast Asia	Colon and rectum cancer	306 (100 to 502)	1,886 (731 to 3,096)	0.23(0.07 to 0.38)	0.61(0.23 to 0.99)
Male	High-income Asia Pacific	Gallbladder and biliary tract cancer	493 (348 to 685)	1,085 (720 to 1,486)	0.56(0.4 to 0.78)	0.48(0.32 to 0.66)
Male	East Asia	Gallbladder and biliary tract cancer	511 (316 to 797)	1,964 (1,059 to 3,126)	0.13(0.08 to 0.2)	0.2(0.11 to 0.31)
Male	South Asia	Gallbladder and biliary tract cancer	134 (78 to 198)	710 (385 to 999)	0.04(0.03 to 0.07)	0.1(0.06 to 0.14)
Male	Central Asia	Gallbladder and biliary tract cancer	22 (15 to 31)	37 (25 to 54)	0.12(0.08 to 0.16)	0.11(0.07 to 0.16)
Male	Southeast Asia	Gallbladder and biliary tract cancer	95 (51 to 146)	451 (209 to 681)	0.08(0.04 to 0.12)	0.15(0.07 to 0.23)
Male	High-income Asia Pacific	Liver cancer	653 (292 to 1,078)	1,280 (525 to 2,160)	0.65(0.29 to 1.07)	0.65(0.27 to 1.09)
Male	East Asia	Liver cancer	1,446 (617 to 2,388)	8,407 (3,239 to 15,441)	0.28(0.12 to 0.47)	0.81(0.31 to 1.47)
Male	South Asia	Liver cancer	119 (49 to 193)	1,192 (471 to 2,025)	0.03(0.01 to 0.06)	0.15(0.06 to 0.26)
Male	Central Asia	Liver cancer	245 (100 to 421)	457 (187 to 836)	1.17(0.49 to 2)	1.19(0.49 to 2.19)
Male	Southeast Asia	Liver cancer	243 (102 to 399)	1,277 (484 to 2,291)	0.16(0.07 to 0.27)	0.38(0.14 to 0.67)
Male	High-income Asia Pacific	Multiple myeloma	46 (-9 to 119)	131 (-29 to 338)	0.05(-0.01 to 0.13)	0.06(-0.01 to 0.16)
Male	East Asia	Multiple myeloma	25 (-4 to 82)	397 (-109 to 1,070)	0.01(0 to 0.02)	0.04(-0.01 to 0.1)
Male	South Asia	Multiple myeloma	31 (-4 to 82)	253 (-63 to 671)	0.01(0 to 0.03)	0.04(-0.01 to 0.09)
Male	Central Asia	Multiple myeloma	4 (-2 to 11)	15 (-6 to 40)	0.02(-0.01 to 0.05)	0.04(-0.02 to 0.1)
Male	Southeast Asia	Multiple myeloma	7 (-1 to 21)	51 (-13 to 148)	0.01(0 to 0.02)	0.02(0 to 0.05)

Sex	Location	Cause	Number of mortality (95%UI)		ASMR (per 100,000 population,95%UI)	
			In 1992	In 2021	In 1992	In 2021
Male	High-income Asia Pacific	Non-Hodgkin lymphoma	113 (39 to 189)	308 (109 to 516)	0.12(0.04 to 0.21)	0.14(0.05 to 0.24)
Male	East Asia	Non-Hodgkin lymphoma	302 (110 to 539)	1,096 (362 to 2,001)	0.06(0.02 to 0.12)	0.11(0.04 to 0.2)
Male	South Asia	Non-Hodgkin lymphoma	133 (50 to 217)	523 (170 to 896)	0.04(0.01 to 0.07)	0.07(0.02 to 0.12)
Male	Central Asia	Non-Hodgkin lymphoma	25 (8 to 41)	35 (11 to 60)	0.11(0.04 to 0.18)	0.09(0.03 to 0.15)
Male	Southeast Asia	Non-Hodgkin lymphoma	67 (23 to 119)	247 (86 to 430)	0.05(0.02 to 0.08)	0.08(0.03 to 0.13)
Male	High-income Asia Pacific	Leukemia	254 (184 to 333)	512 (365 to 693)	0.27(0.19 to 0.35)	0.26(0.18 to 0.35)
Male	East Asia	Leukemia	1,138 (713 to 1,669)	2,565 (1,563 to 3,863)	0.22(0.14 to 0.33)	0.27(0.16 to 0.4)
Male	South Asia	Leukemia	292 (183 to 421)	946 (622 to 1,342)	0.08(0.05 to 0.12)	0.13(0.08 to 0.18)
Male	Central Asia	Leukemia	79 (60 to 101)	119 (87 to 159)	0.33(0.25 to 0.43)	0.3(0.22 to 0.4)
Male	Southeast Asia	Leukemia	222 (143 to 317)	646 (431 to 947)	0.15(0.1 to 0.22)	0.2(0.14 to 0.3)
Male	High-income Asia Pacific	Kidney cancer	204 (78 to 334)	676 (261 to 1,091)	0.22(0.08 to 0.36)	0.32(0.12 to 0.51)
Male	East Asia	Kidney cancer	388 (153 to 639)	2,497 (953 to 4,365)	0.1(0.04 to 0.16)	0.25(0.1 to 0.44)
Male	South Asia	Kidney cancer	88 (32 to 142)	584 (219 to 960)	0.03(0.01 to 0.04)	0.08(0.03 to 0.13)
Male	Central Asia	Kidney cancer	86 (32 to 147)	261 (101 to 437)	0.41(0.15 to 0.7)	0.69(0.27 to 1.16)
Male	Southeast Asia	Kidney cancer	76 (28 to 125)	386 (143 to 636)	0.06(0.02 to 0.09)	0.12(0.04 to 0.2)
Male	High-income Asia Pacific	Thyroid cancer	35 (24 to 46)	87 (60 to 117)	0.04(0.03 to 0.05)	0.04(0.03 to 0.05)
Male	East Asia	Thyroid cancer	100 (66 to 152)	470 (291 to 671)	0.03(0.02 to 0.04)	0.05(0.03 to 0.07)
Male	South Asia	Thyroid cancer	49 (34 to 70)	236 (170 to 313)	0.02(0.01 to 0.02)	0.03(0.02 to 0.04)
Male	Central Asia	Thyroid cancer	11 (9 to 15)	16 (11 to 21)	0.05(0.04 to 0.07)	0.04(0.03 to 0.06)
Male	Southeast Asia	Thyroid cancer	31 (22 to 43)	132 (90 to 180)	0.02(0.02 to 0.03)	0.04(0.03 to 0.06)
Female	High-income Asia Pacific	Breast cancer	307 (-8 to 621)	1,007 (-25 to 2,088)	0.25(-0.01 to 0.52)	0.42(-0.01 to 0.85)
Female	East Asia	Breast cancer	1,244 (-32 to 2,644)	6,003 (-191 to 12,971)	0.27(-0.01 to 0.57)	0.5(-0.02 to 1.08)

Sex	Location	Cause	Number of mortality (95%UI)		ASMR (per 100,000 population,95%UI)	
			In 1992	In 2021	In 1992	In 2021
Female	South Asia	Breast cancer	320 (-63 to 699)	2,698 (-233 to 5,467)	0.14(-0.01 to 0.29)	0.38(-0.02 to 0.77)
Female	Central Asia	Breast cancer	313 (-11 to 608)	539 (-20 to 1,053)	1.05(-0.04 to 2.03)	1.15(-0.04 to 2.24)
Female	Southeast Asia	Breast cancer	519 (-13 to 1,072)	2,990 (-78 to 6,339)	0.35(-0.01 to 0.72)	0.8(-0.02 to 1.7)
Female	High-income Asia Pacific	Ovarian cancer	92 (-9 to 204)	253 (22 to 508)	0.08(-0.01 to 0.17)	0.12(0.01 to 0.24)
Female	East Asia	Ovarian cancer	174 (-59 to 456)	1,814 (356 to 3,763)	0.04(-0.01 to 0.09)	0.16(0.03 to 0.33)
Female	South Asia	Ovarian cancer	110 (-14 to 245)	1,303 (208 to 2,414)	0.03(-0.01 to 0.07)	0.16(0.03 to 0.3)
Female	Central Asia	Ovarian cancer	92 (20 to 168)	255 (62 to 459)	0.33(0.07 to 0.59)	0.53(0.13 to 0.95)
Female	Southeast Asia	Ovarian cancer	87 (-3 to 189)	763 (142 to 1,449)	0.05(0 to 0.11)	0.2(0.04 to 0.38)
Female	High-income Asia Pacific	Uterine cancer	314 (231 to 421)	733 (499 to 983)	0.26(0.19 to 0.35)	0.31(0.22 to 0.42)
Female	East Asia	Uterine cancer	1,327 (845 to 1,980)	3,778 (2,322 to 5,853)	0.27(0.17 to 0.4)	0.33(0.2 to 0.51)
Female	South Asia	Uterine cancer	332 (228 to 461)	1,799 (1,195 to 2,637)	0.11(0.08 to 0.16)	0.23(0.16 to 0.35)
Female	Central Asia	Uterine cancer	361 (256 to 477)	516 (361 to 685)	1.27(0.9 to 1.68)	1.11(0.77 to 1.47)
Female	Southeast Asia	Uterine cancer	355 (229 to 495)	1,619 (993 to 2,247)	0.23(0.15 to 0.32)	0.43(0.27 to 0.6)
Female	High-income Asia Pacific	Colon and rectum cancer	813 (303 to 1,312)	2,127 (815 to 3,482)	0.67(0.25 to 1.08)	0.66(0.26 to 1.06)
Female	East Asia	Colon and rectum cancer	2,006 (684 to 3,447)	8,689 (3,469 to 15,102)	0.44(0.15 to 0.76)	0.76(0.31 to 1.33)
Female	South Asia	Colon and rectum cancer	342 (117 to 565)	1,698 (683 to 2,740)	0.11(0.04 to 0.19)	0.22(0.09 to 0.35)
Female	Central Asia	Colon and rectum cancer	288 (119 to 454)	421 (183 to 663)	1.02(0.42 to 1.61)	0.94(0.41 to 1.49)
Female	Southeast Asia	Colon and rectum cancer	430 (161 to 693)	2,172 (886 to 3,595)	0.29(0.11 to 0.47)	0.62(0.25 to 1.02)
Female	High-income Asia Pacific	Gallbladder and biliary tract cancer	721 (498 to 984)	1,163 (675 to 1,657)	0.58(0.4 to 0.8)	0.33(0.21 to 0.46)
Female	East Asia	Gallbladder and biliary tract cancer	639 (398 to 984)	2,238 (1,253 to 3,479)	0.14(0.09 to 0.22)	0.19(0.11 to 0.3)
Female	South Asia	Gallbladder and biliary tract cancer	317 (202 to 472)	1,699 (999 to 2,443)	0.11(0.07 to 0.17)	0.23(0.13 to 0.33)
Female	Central Asia	Gallbladder and biliary tract cancer	55 (36 to 78)	75 (51 to 106)	0.2(0.13 to 0.28)	0.17(0.11 to 0.24)

Sex	Location	Cause	Number of mortality (95%UI)		ASMR (per 100,000 population,95%UI)	
			In 1992	In 2021	In 1992	In 2021
Female	Southeast Asia	Gallbladder and biliary tract cancer	143 (92 to 209)	519 (323 to 777)	0.1(0.07 to 0.15)	0.15(0.09 to 0.23)
Female	High-income Asia Pacific	Liver cancer	270 (118 to 436)	760 (292 to 1,284)	0.22(0.1 to 0.35)	0.25(0.1 to 0.42)
Female	East Asia	Liver cancer	631 (267 to 1,081)	4,791 (1,927 to 8,696)	0.13(0.06 to 0.23)	0.41(0.17 to 0.75)
Female	South Asia	Liver cancer	82 (34 to 133)	800 (321 to 1,320)	0.03(0.01 to 0.04)	0.1(0.04 to 0.17)
Female	Central Asia	Liver cancer	232 (96 to 385)	421 (174 to 748)	0.82(0.34 to 1.36)	0.93(0.38 to 1.63)
Female	Southeast Asia	Liver cancer	154 (62 to 251)	814 (312 to 1,469)	0.1(0.04 to 0.17)	0.23(0.09 to 0.41)
Female	High-income Asia Pacific	Multiple myeloma	56 (-13 to 143)	137 (-38 to 372)	0.04(-0.01 to 0.12)	0.04(-0.01 to 0.12)
Female	East Asia	Multiple myeloma	25 (-7 to 84)	352 (-123 to 963)	0.01(0 to 0.02)	0.03(-0.01 to 0.08)
Female	South Asia	Multiple myeloma	30 (-7 to 86)	264 (-85 to 723)	0.01(0 to 0.03)	0.04(-0.01 to 0.1)
Female	Central Asia	Multiple myeloma	6 (-2 to 14)	21 (-10 to 53)	0.02(-0.01 to 0.05)	0.04(-0.02 to 0.11)
Female	Southeast Asia	Multiple myeloma	11 (-3 to 29)	78 (-31 to 231)	0.01(0 to 0.02)	0.02(-0.01 to 0.06)
Female	High-income Asia Pacific	Non-Hodgkin lymphoma	87 (31 to 147)	257 (78 to 454)	0.07(0.03 to 0.12)	0.08(0.03 to 0.14)
Female	East Asia	Non-Hodgkin lymphoma	234 (85 to 411)	741 (223 to 1,339)	0.05(0.02 to 0.09)	0.07(0.02 to 0.12)
Female	South Asia	Non-Hodgkin lymphoma	87 (31 to 150)	455 (155 to 782)	0.03(0.01 to 0.05)	0.06(0.02 to 0.1)
Female	Central Asia	Non-Hodgkin lymphoma	19 (6 to 33)	34 (11 to 59)	0.07(0.02 to 0.11)	0.07(0.02 to 0.12)
Female	Southeast Asia	Non-Hodgkin lymphoma	63 (24 to 110)	255 (88 to 451)	0.04(0.02 to 0.07)	0.07(0.02 to 0.13)
Female	High-income Asia Pacific	Leukemia	200 (141 to 264)	347 (215 to 481)	0.17(0.12 to 0.23)	0.13(0.09 to 0.18)
Female	East Asia	Leukemia	1,028 (702 to 1,487)	1,989 (1,208 to 2,880)	0.19(0.13 to 0.28)	0.19(0.11 to 0.27)
Female	South Asia	Leukemia	263 (176 to 365)	841 (593 to 1,210)	0.08(0.05 to 0.11)	0.11(0.07 to 0.15)
Female	Central Asia	Leukemia	79 (59 to 102)	113 (82 to 152)	0.27(0.2 to 0.34)	0.24(0.17 to 0.32)
Female	Southeast Asia	Leukemia	304 (206 to 427)	818 (573 to 1,136)	0.18(0.13 to 0.25)	0.23(0.16 to 0.32)
Female	High-income Asia Pacific	Kidney cancer	111 (42 to 183)	397 (138 to 663)	0.09(0.03 to 0.15)	0.12(0.04 to 0.2)

Sex	Location	Cause	Number of mortality (95%UI)		ASMR (per 100,000 population,95%UI)	
			In 1992	In 2021	In 1992	In 2021
Female	East Asia	Kidney cancer	225 (85 to 377)	1,302 (515 to 2,304)	0.05(0.02 to 0.09)	0.11(0.05 to 0.2)
Female	South Asia	Kidney cancer	40 (15 to 66)	319 (114 to 532)	0.01(0.01 to 0.02)	0.04(0.02 to 0.07)
Female	Central Asia	Kidney cancer	91 (36 to 150)	164 (67 to 265)	0.32(0.13 to 0.53)	0.35(0.15 to 0.57)
Female	Southeast Asia	Kidney cancer	52 (19 to 84)	267 (100 to 443)	0.04(0.01 to 0.06)	0.08(0.03 to 0.13)
Female	High-income Asia Pacific	Thyroid cancer	82 (55 to 111)	192 (112 to 268)	0.07(0.04 to 0.09)	0.06(0.04 to 0.08)
Female	East Asia	Thyroid cancer	209 (142 to 299)	443 (285 to 636)	0.05(0.03 to 0.07)	0.04(0.03 to 0.06)
Female	South Asia	Thyroid cancer	109 (74 to 157)	469 (319 to 633)	0.04(0.02 to 0.05)	0.06(0.04 to 0.08)
Female	Central Asia	Thyroid cancer	26 (20 to 33)	38 (28 to 49)	0.09(0.07 to 0.12)	0.08(0.06 to 0.11)
Female	Southeast Asia	Thyroid cancer	103 (73 to 140)	352 (241 to 474)	0.07(0.05 to 0.1)	0.1(0.07 to 0.14)

Table S5. The changes in deathes of total cancer attributable to high BMI associated with population ageing, population growth, and epidemiological change between 1992 and 2021 in Asia, five GBD regions, and 34 countries and territories.

Location	Overall difference	Aging	Population	Epidemiological change	Percent change of Aging	Percent change of Population	Percent change of Epidemiological change	Overall percent change
Asia	93,784.42	23,876.20	34,555.23	35,352.98	79.83	115.54	118.20	313.57
**Five GBD regions**								
High-income Asia Pacific	7,749.59	5,108.32	1,711.01	930.26	95.76	32.07	17.44	145.27
East Asia	48,313.64	15,945.70	11,090.22	21,277.73	122.11	84.93	162.94	369.99
South Asia	15,057.90	1,770.05	5,940.39	7,347.46	57.98	194.59	240.68	493.26
Central Asia	1,742.20	266.06	1,395.32	80.82	11.83	62.02	3.59	77.44
Southeast Asia	12,490.95	2,371.66	4,650.76	5,468.53	74.16	145.43	171.01	390.60

Table S6. The changes in deathes of 11 cancer subtypes attributable to high BMI associated with population ageing, population growth, and epidemiological change between 1992 and 2021 in Asia.

Sex	Cause	Overall difference	Aging	Population	Epidemiological change	Percent change of Aging	Percent change of Population	Percent change of Epidemiological change	Overall percent change
Male	Kidney cancer	4,076.67	936.98	1,290.52	1,849.17	95.19	131.11	187.86	414.16
Male	Thyroid cancer	773.12	199.00	275.22	298.90	82.89	114.64	124.50	322.03
Male	Liver cancer	10,806.52	2,070.22	3,571.07	5,165.22	72.54	125.12	180.98	378.64
Male	Colon and rectum cancer	14,869.85	3,657.86	4,999.63	6,212.36	88.81	121.39	150.83	361.03
Male	Gallbladder and biliary tract cancer	3,126.50	1,059.77	1,294.25	772.48	81.74	99.83	59.58	241.15
Male	Multiple myeloma	898.80	191.46	252.27	455.07	120.41	158.66	286.20	565.26
Male	Leukemia	3,385.81	894.61	1,858.45	632.75	40.09	83.28	28.35	151.72
Male	Non-Hodgkin lymphoma	1,800.07	456.48	738.96	604.63	62.21	100.71	82.40	245.32
Female	Kidney cancer	2,120.11	583.76	759.67	776.68	95.38	124.12	126.90	346.40
Female	Thyroid cancer	1,033.28	356.13	524.77	152.38	64.18	94.58	27.46	186.22
Female	Ovarian cancer	4,213.41	724.36	1,232.21	2,256.84	99.81	169.79	310.98	580.59
Female	Breast cancer	11,351.08	3,106.04	3,901.34	4,343.70	103.77	130.34	145.12	379.23
Female	Uterine cancer	6,304.59	1,810.99	2,841.13	1,652.47	65.05	102.05	59.36	226.46
Female	Liver cancer	6,722.64	1,499.18	2,132.92	3,090.54	101.61	144.57	209.47	455.65
Female	Colon and rectum cancer	12,143.02	3,721.41	4,882.78	3,538.83	84.61	111.01	80.46	276.08
Female	Gallbladder and biliary tract cancer	3,988.39	1,550.08	1,918.08	520.23	79.17	97.97	26.57	203.72

Female	Multiple myeloma	846.04	193.58	260.56	391.90	113.34	152.57	229.46	495.37
Female	Leukemia	2,645.96	807.51	1,740.34	98.11	38.21	82.34	4.64	125.19
Female	Non-Hodgkin lymphoma	1,436.08	390.69	610.41	434.97	68.18	106.52	75.90	250.60

Table S7. Proportion of deaths for total cancer attributable to high BMI across 16 age groups in 1992 and 2021 within Asia and five GBD regions.

Age	Year	Asia	High-income Asia Pacific	East Asia	South Asia	Southeast Asia	Central Asia
20 to 24	1992	1.19	0.3	1.61	1.45	1.4	0.56
20 to 24	2021	0.35	0.05	0.26	0.58	0.5	0.29
25 to 29	1992	1.44	0.38	1.94	1.63	1.88	0.82
25 to 29	2021	0.55	0.08	0.49	0.74	0.83	0.56
30 to 34	1992	1.79	0.61	2.38	1.61	2.68	1
95 plus	1992	0.2	0.42	0.06	0.1	0.13	0.23
95 plus	2021	0.94	5.15	0.38	0.19	0.32	0.21
30 to 34	2021	1.1	0.14	1.3	0.92	1.36	0.83
35 to 39	1992	3.01	1.06	4.49	1.77	4.02	1.3
35 to 39	2021	1.67	0.31	1.93	1.27	2.33	1.19
40 to 44	1992	4.25	2.45	6.08	1.84	5.42	2.12
40 to 44	2021	2.51	0.71	2.81	1.7	3.82	2.01
45 to 49	1992	5.24	3.72	6.58	3.52	7.16	2.49
45 to 49	2021	4.38	1.61	5.01	3.09	6.01	3.55
50 to 54	1992	10.81	7.87	10.49	15	14.3	12.68
50 to 54	2021	10.29	3.67	10.28	12.75	13.48	10.33
55 to 59	1992	13.54	11.7	13.46	17.42	15.57	14.89
55 to 59	2021	12.58	5.36	12.19	16.84	15.8	15.99
60 to 64	1992	14.34	14.28	13.4	16.23	14.68	19.82
60 to 64	2021	12.55	7.22	11.68	15.58	15.72	19.57
65 to 69	1992	13.89	14.64	13.02	14.35	12.4	17.61

Age	Year	Asia	High-income Asia Pacific	East Asia	South Asia	Southeast Asia	Central Asia
65 to 69	2021	14.72	9.83	15.74	16.11	14.21	17.55
70 to 74	1992	11.81	12.86	11.44	11.64	9.09	11.01
70 to 74	2021	13.22	13.69	13.85	13.34	10.8	12
75 to 79	1992	9.18	12.68	8.41	7.38	5.84	8.13
75 to 79	2021	10.17	14.18	10.51	8.75	7.34	6.85
80 to 84	1992	5.41	9.42	4.13	3.74	3.14	4.76
80 to 84	2021	7.15	13.59	7.04	4.77	4.13	6.29
85 to 89	1992	2.89	5.4	2.04	1.79	1.74	1.82
85 to 89	2021	5.21	14.05	4.78	2.45	2.32	1.96
90 to 94	1992	0.99	2.19	0.47	0.53	0.54	0.75
90 to 94	2021	2.62	10.35	1.76	0.92	1.04	0.83

Table S8. The local drift of mortality from 1992 to 2021 for total cancer attributable to high BMI across 16 age groups within Asia and five GBD regions.

Cause	Location	Age groups	Local drift (%/year)
Total cancers	Asia	20 to 24	0.73 (0.37 to 1.08)
Total cancers	Asia	25 to 29	1.01 (0.77 to 1.24)
Total cancers	Asia	30 to 34	1.24 (1.06 to 1.41)
Total cancers	Asia	35 to 39	1.23 (1.09 to 1.38)
Total cancers	Asia	40 to 44	1.38 (1.27 to 1.50)
Total cancers	Asia	45 to 49	1.67 (1.58 to 1.76)
Total cancers	Asia	50 to 54	1.88 (1.80 to 1.95)
Total cancers	Asia	55 to 59	1.88 (1.81 to 1.95)
Total cancers	Asia	60 to 64	1.89 (1.83 to 1.95)
Total cancers	Asia	65 to 69	1.82 (1.76 to 1.89)
Total cancers	Asia	70 to 74	1.76 (1.69 to 1.83)
Total cancers	Asia	75 to 79	1.81 (1.73 to 1.90)
Total cancers	Asia	80 to 84	1.93 (1.83 to 2.04)
Total cancers	Asia	85 to 89	2.22 (2.06 to 2.37)
Total cancers	Asia	90 to 94	2.56 (2.27 to 2.85)
Total cancers	Asia	95 plus	3.03 (2.33 to 3.74)
Total cancers	High-income Asia Pacific	20 to 24	-1.63 (-4.23 to 1.04)
Total cancers	High-income Asia Pacific	25 to 29	-1.30 (-2.90 to 0.33)
Total cancers	High-income Asia Pacific	30 to 34	-1.36 (-2.47 to -0.23)
Total cancers	High-income Asia Pacific	35 to 39	-1.34 (-2.11 to -0.57)
Total cancers	High-income Asia Pacific	40 to 44	-1.28 (-1.81 to -0.74)

Cause	Location	Age groups	Local drift (%/year)
Total cancers	High-income Asia Pacific	45 to 49	-0.96 (-1.33 to -0.59)
Total cancers	High-income Asia Pacific	50 to 54	-0.70 (-0.97 to -0.42)
Total cancers	High-income Asia Pacific	55 to 59	-0.54 (-0.76 to -0.32)
Total cancers	High-income Asia Pacific	60 to 64	-0.53 (-0.72 to -0.35)
Total cancers	High-income Asia Pacific	65 to 69	-0.42 (-0.59 to -0.26)
Total cancers	High-income Asia Pacific	70 to 74	-0.22 (-0.38 to -0.06)
Total cancers	High-income Asia Pacific	75 to 79	0.21 (0.04 to 0.38)
Total cancers	High-income Asia Pacific	80 to 84	0.71 (0.52 to 0.91)
Total cancers	High-income Asia Pacific	85 to 89	1.32 (1.06 to 1.57)
Total cancers	High-income Asia Pacific	90 to 94	1.93 (1.50 to 2.37)
Total cancers	High-income Asia Pacific	95 plus	2.71 (1.65 to 3.78)
Total cancers	Southeast Asia	20 to 24	1.00 (0.14 to 1.87)
Total cancers	Southeast Asia	25 to 29	1.08 (0.50 to 1.66)
Total cancers	Southeast Asia	30 to 34	1.27 (0.83 to 1.70)
Total cancers	Southeast Asia	35 to 39	1.50 (1.16 to 1.84)
Total cancers	Southeast Asia	40 to 44	1.68 (1.40 to 1.96)
Total cancers	Southeast Asia	45 to 49	1.82 (1.59 to 2.05)
Total cancers	Southeast Asia	50 to 54	2.01 (1.81 to 2.20)
Total cancers	Southeast Asia	55 to 59	2.22 (2.04 to 2.40)
Total cancers	Southeast Asia	60 to 64	2.53 (2.35 to 2.72)
Total cancers	Southeast Asia	65 to 69	2.87 (2.67 to 3.07)
Total cancers	Southeast Asia	70 to 74	3.10 (2.86 to 3.34)
Total cancers	Southeast Asia	75 to 79	3.14 (2.82 to 3.46)

Cause	Location	Age groups	Local drift (%/year)
Total cancers	Southeast Asia	80 to 84	3.06 (2.62 to 3.51)
Total cancers	Southeast Asia	85 to 89	2.94 (2.29 to 3.59)
Total cancers	Southeast Asia	90 to 94	3.15 (1.98 to 4.33)
Total cancers	Southeast Asia	95 plus	3.62 (0.89 to 6.43)
Total cancers	East Asia	20 to 24	1.61 (0.91 to 2.32)
Total cancers	East Asia	25 to 29	1.91 (1.47 to 2.34)
Total cancers	East Asia	30 to 34	2.13 (1.82 to 2.44)
Total cancers	East Asia	35 to 39	1.98 (1.73 to 2.22)
Total cancers	East Asia	40 to 44	1.82 (1.63 to 2.02)
Total cancers	East Asia	45 to 49	1.90 (1.74 to 2.06)
Total cancers	East Asia	50 to 54	2.14 (2.00 to 2.27)
Total cancers	East Asia	55 to 59	2.24 (2.11 to 2.36)
Total cancers	East Asia	60 to 64	2.44 (2.31 to 2.56)
Total cancers	East Asia	65 to 69	2.51 (2.39 to 2.64)
Total cancers	East Asia	70 to 74	2.57 (2.43 to 2.71)
Total cancers	East Asia	75 to 79	2.68 (2.51 to 2.85)
Total cancers	East Asia	80 to 84	2.83 (2.59 to 3.07)
Total cancers	East Asia	85 to 89	2.75 (2.37 to 3.14)
Total cancers	East Asia	90 to 94	2.47 (1.68 to 3.27)
Total cancers	East Asia	95 plus	2.11 (-0.26 to 4.54)
Total cancers	South Asia	20 to 24	0.92 (0.13 to 1.71)
Total cancers	South Asia	25 to 29	1.54 (0.94 to 2.15)
Total cancers	South Asia	30 to 34	2.03 (1.51 to 2.55)

Cause	Location	Age groups	Local drift (%/year)
Total cancers	South Asia	35 to 39	2.58 (2.13 to 3.03)
Total cancers	South Asia	40 to 44	3.04 (2.66 to 3.42)
Total cancers	South Asia	45 to 49	3.22 (2.93 to 3.52)
Total cancers	South Asia	50 to 54	3.28 (3.07 to 3.49)
Total cancers	South Asia	55 to 59	3.33 (3.14 to 3.51)
Total cancers	South Asia	60 to 64	3.21 (3.04 to 3.39)
Total cancers	South Asia	65 to 69	3.22 (3.03 to 3.40)
Total cancers	South Asia	70 to 74	3.16 (2.94 to 3.38)
Total cancers	South Asia	75 to 79	3.17 (2.88 to 3.46)
Total cancers	South Asia	80 to 84	3.20 (2.79 to 3.62)
Total cancers	South Asia	85 to 89	3.28 (2.63 to 3.92)
Total cancers	South Asia	90 to 94	3.31 (2.09 to 4.54)
Total cancers	South Asia	95 plus	3.26 (0.24 to 6.37)
Total cancers	Central Asia	20 to 24	-0.47 (-2.42 to 1.52)
Total cancers	Central Asia	25 to 29	-0.54 (-1.84 to 0.77)
Total cancers	Central Asia	30 to 34	-0.37 (-1.39 to 0.65)
Total cancers	Central Asia	35 to 39	-0.31 (-1.13 to 0.52)
Total cancers	Central Asia	40 to 44	-0.38 (-1.01 to 0.26)
Total cancers	Central Asia	45 to 49	-0.55 (-1.03 to -0.07)
Total cancers	Central Asia	50 to 54	-0.59 (-0.93 to -0.24)
Total cancers	Central Asia	55 to 59	-0.37 (-0.65 to -0.09)
Total cancers	Central Asia	60 to 64	-0.04 (-0.30 to 0.22)
Total cancers	Central Asia	65 to 69	0.25 (-0.02 to 0.51)

Cause	Location	Age groups	Local drift (%/year)
Total cancers	Central Asia	70 to 74	0.53 (0.21 to 0.86)
Total cancers	Central Asia	75 to 79	1.03 (0.61 to 1.45)
Total cancers	Central Asia	80 to 84	1.50 (0.94 to 2.07)
Total cancers	Central Asia	85 to 89	1.83 (0.95 to 2.71)
Total cancers	Central Asia	90 to 94	1.55 (0.13 to 2.99)
Total cancers	Central Asia	95 plus	0.40 (-2.46 to 3.35)

Table S9 Age effects on mortality of total cancer attributable to high BMI across Asia and five GBD regions.

Cause	Location	Age groups	Rate (per 100,000 population)
Total cancers	Asia	20 to 24	0.10 (0.10 to 0.11)
Total cancers	Asia	25 to 29	0.16 (0.15 to 0.16)
Total cancers	Asia	30 to 34	0.28 (0.27 to 0.29)
Total cancers	Asia	35 to 39	0.51 (0.50 to 0.52)
Total cancers	Asia	40 to 44	0.95 (0.93 to 0.98)
Total cancers	Asia	45 to 49	1.77 (1.73 to 1.81)
Total cancers	Asia	50 to 54	4.54 (4.43 to 4.65)
Total cancers	Asia	55 to 59	6.97 (6.80 to 7.15)
Total cancers	Asia	60 to 64	9.81 (9.57 to 10.07)
Total cancers	Asia	65 to 69	13.96 (13.60 to 14.33)
Total cancers	Asia	70 to 74	19.42 (18.91 to 19.96)
Total cancers	Asia	75 to 79	26.31 (25.58 to 27.05)
Total cancers	Asia	80 to 84	32.92 (31.96 to 33.90)
Total cancers	Asia	85 to 89	52.44 (50.81 to 54.13)
Total cancers	Asia	90 to 94	79.29 (76.42 to 82.26)
Total cancers	Asia	95 plus	107.14 (101.81 to 112.75)
Total cancers	High-income Asia Pacific	20 to 24	0.09 (0.07 to 0.13)
Total cancers	High-income Asia Pacific	25 to 29	0.13 (0.10 to 0.17)
Total cancers	High-income Asia Pacific	30 to 34	0.21 (0.18 to 0.25)
Total cancers	High-income Asia Pacific	35 to 39	0.38 (0.33 to 0.43)
Total cancers	High-income Asia Pacific	40 to 44	0.75 (0.67 to 0.84)
Total cancers	High-income Asia Pacific	45 to 49	1.40 (1.26 to 1.55)

Cause	Location	Age groups	Rate (per 100,000 population)
Total cancers	High-income Asia Pacific	50 to 54	3.08 (2.75 to 3.45)
Total cancers	High-income Asia Pacific	55 to 59	4.66 (4.16 to 5.22)
Total cancers	High-income Asia Pacific	60 to 64	6.55 (5.84 to 7.33)
Total cancers	High-income Asia Pacific	65 to 69	8.98 (8.01 to 10.06)
Total cancers	High-income Asia Pacific	70 to 74	11.44 (10.20 to 12.83)
Total cancers	High-income Asia Pacific	75 to 79	15.41 (13.73 to 17.30)
Total cancers	High-income Asia Pacific	80 to 84	18.80 (16.73 to 21.13)
Total cancers	High-income Asia Pacific	85 to 89	28.26 (25.11 to 31.80)
Total cancers	High-income Asia Pacific	90 to 94	41.76 (37.00 to 47.13)
Total cancers	High-income Asia Pacific	95 plus	51.60 (45.37 to 58.70)
Total cancers	East Asia	20 to 24	0.13 (0.12 to 0.15)
Total cancers	East Asia	25 to 29	0.20 (0.19 to 0.22)
Total cancers	East Asia	30 to 34	0.40 (0.38 to 0.43)
Total cancers	East Asia	35 to 39	0.77 (0.73 to 0.80)
Total cancers	East Asia	40 to 44	1.45 (1.40 to 1.51)
Total cancers	East Asia	45 to 49	2.51 (2.42 to 2.60)
Total cancers	East Asia	50 to 54	5.11 (4.90 to 5.32)
Total cancers	East Asia	55 to 59	7.68 (7.36 to 8.01)
Total cancers	East Asia	60 to 64	11.20 (10.72 to 11.70)
Total cancers	East Asia	65 to 69	16.16 (15.45 to 16.92)
Total cancers	East Asia	70 to 74	23.42 (22.34 to 24.55)
Total cancers	East Asia	75 to 79	32.74 (31.17 to 34.39)
Total cancers	East Asia	80 to 84	40.80 (38.70 to 43.00)

Cause	Location	Age groups	Rate (per 100,000 population)
Total cancers	East Asia	85 to 89	65.01 (61.34 to 68.90)
Total cancers	East Asia	90 to 94	86.01 (79.73 to 92.79)
Total cancers	East Asia	95 plus	91.98 (79.62 to 106.27)
Total cancers	Southeast Asia	20 to 24	0.10 (0.09 to 0.12)
Total cancers	Southeast Asia	25 to 29	0.18 (0.16 to 0.20)
Total cancers	Southeast Asia	30 to 34	0.33 (0.30 to 0.35)
Total cancers	Southeast Asia	35 to 39	0.62 (0.58 to 0.66)
Total cancers	Southeast Asia	40 to 44	1.15 (1.09 to 1.22)
Total cancers	Southeast Asia	45 to 49	2.14 (2.04 to 2.26)
Total cancers	Southeast Asia	50 to 54	5.64 (5.32 to 5.98)
Total cancers	Southeast Asia	55 to 59	8.29 (7.80 to 8.81)
Total cancers	Southeast Asia	60 to 64	11.04 (10.36 to 11.77)
Total cancers	Southeast Asia	65 to 69	14.84 (13.89 to 15.86)
Total cancers	Southeast Asia	70 to 74	18.94 (17.66 to 20.31)
Total cancers	Southeast Asia	75 to 79	23.67 (21.98 to 25.50)
Total cancers	Southeast Asia	80 to 84	25.29 (23.26 to 27.50)
Total cancers	Southeast Asia	85 to 89	34.64 (31.40 to 38.22)
Total cancers	Southeast Asia	90 to 94	48.72 (42.74 to 55.54)
Total cancers	Southeast Asia	95 plus	61.82 (50.14 to 76.22)
Total cancers	South Asia	20 to 24	0.05 (0.04 to 0.05)
Total cancers	South Asia	25 to 29	0.06 (0.06 to 0.07)
Total cancers	South Asia	30 to 34	0.09 (0.08 to 0.10)
Total cancers	South Asia	35 to 39	0.15 (0.13 to 0.16)

Cause	Location	Age groups	Rate (per 100,000 population)
Total cancers	South Asia	40 to 44	0.22 (0.20 to 0.24)
Total cancers	South Asia	45 to 49	0.55 (0.51 to 0.59)
Total cancers	South Asia	50 to 54	3.15 (2.92 to 3.41)
Total cancers	South Asia	55 to 59	5.49 (5.07 to 5.96)
Total cancers	South Asia	60 to 64	7.41 (6.82 to 8.05)
Total cancers	South Asia	65 to 69	11.20 (10.29 to 12.19)
Total cancers	South Asia	70 to 74	15.47 (14.18 to 16.87)
Total cancers	South Asia	75 to 79	19.04 (17.39 to 20.83)
Total cancers	South Asia	80 to 84	20.84 (18.92 to 22.96)
Total cancers	South Asia	85 to 89	29.31 (26.31 to 32.65)
Total cancers	South Asia	90 to 94	41.47 (36.20 to 47.51)
Total cancers	South Asia	95 plus	46.84 (37.18 to 59.03)
Total cancers	Central Asia	20 to 24	0.19 (0.14 to 0.25)
Total cancers	Central Asia	25 to 29	0.31 (0.25 to 0.39)
Total cancers	Central Asia	30 to 34	0.42 (0.34 to 0.51)
Total cancers	Central Asia	35 to 39	0.69 (0.58 to 0.82)
Total cancers	Central Asia	40 to 44	1.36 (1.18 to 1.57)
Total cancers	Central Asia	45 to 49	2.75 (2.42 to 3.12)
Total cancers	Central Asia	50 to 54	9.05 (7.88 to 10.40)
Total cancers	Central Asia	55 to 59	13.44 (11.67 to 15.46)
Total cancers	Central Asia	60 to 64	18.91 (16.39 to 21.82)
Total cancers	Central Asia	65 to 69	24.41 (21.11 to 28.22)
Total cancers	Central Asia	70 to 74	29.97 (25.86 to 34.72)

Cause	Location	Age groups	Rate (per 100,000 population)
Total cancers	Central Asia	75 to 79	31.71 (27.26 to 36.88)
Total cancers	Central Asia	80 to 84	30.43 (25.93 to 35.71)
Total cancers	Central Asia	85 to 89	24.60 (20.39 to 29.68)
Total cancers	Central Asia	90 to 94	27.76 (21.72 to 35.47)
Total cancers	Central Asia	95 plus	31.61 (21.45 to 46.59)

Table S10. Period effects on mortality of total cancer attributable to high BMI across Asia and five GBD regions.

Location	Cause	Rei	Period	Rate Ratio
Asia	Total cancers	High body-mass index	1992 to 1996	1.00 (1.00 to1.00)
Asia	Total cancers	High body-mass index	1997 to 2001	1.10 (1.08 to1.11)
Asia	Total cancers	High body-mass index	2002 to 2006	1.19 (1.17 to1.20)
Asia	Total cancers	High body-mass index	2007 to 2011	1.28 (1.27 to1.30)
Asia	Total cancers	High body-mass index	2012 to 2016	1.40 (1.38 to1.42)
Asia	Total cancers	High body-mass index	2017 to 2021	1.56 (1.53 to1.58)
High-income Asia Pacific	Total cancers	High body-mass index	1992 to 1996	1.00 (1.00 to1.00)
High-income Asia Pacific	Total cancers	High body-mass index	1997 to 2001	1.00 (0.96 to1.03)
High-income Asia Pacific	Total cancers	High body-mass index	2002 to 2006	0.95 (0.92 to0.99)
High-income Asia Pacific	Total cancers	High body-mass index	2007 to 2011	0.93 (0.89 to0.97)
High-income Asia Pacific	Total cancers	High body-mass index	2012 to 2016	0.93 (0.88 to0.97)
High-income Asia Pacific	Total cancers	High body-mass index	2017 to 2021	0.93 (0.88 to0.98)
East Asia	Total cancers	High body-mass index	1992 to 1996	1.00 (1.00 to1.00)
East Asia	Total cancers	High body-mass index	1997 to 2001	1.12 (1.08 to1.15)
East Asia	Total cancers	High body-mass index	2002 to 2006	1.24 (1.21 to1.28)
East Asia	Total cancers	High body-mass index	2007 to 2011	1.37 (1.34 to1.41)
East Asia	Total cancers	High body-mass index	2012 to 2016	1.54 (1.49 to1.59)
East Asia	Total cancers	High body-mass index	2017 to 2021	1.78 (1.72 to1.84)
Southeast Asia	Total cancers	High body-mass index	1992 to 1996	1.00 (1.00 to1.00)
Southeast Asia	Total cancers	High body-mass index	1997 to 2001	1.16 (1.11 to1.21)
Southeast Asia	Total cancers	High body-mass index	2002 to 2006	1.32 (1.27 to1.38)
Southeast Asia	Total cancers	High body-mass index	2007 to 2011	1.47 (1.41 to1.54)

Location	Cause	Rei	Period	Rate Ratio
Southeast Asia	Total cancers	High body-mass index	2012 to 2016	1.62 (1.55 to1.69)
Southeast Asia	Total cancers	High body-mass index	2017 to 2021	1.80 (1.72 to1.88)
South Asia	Total cancers	High body-mass index	1992 to 1996	1.00 (1.00 to1.00)
South Asia	Total cancers	High body-mass index	1997 to 2001	1.16 (1.11 to1.22)
South Asia	Total cancers	High body-mass index	2002 to 2006	1.34 (1.29 to1.40)
South Asia	Total cancers	High body-mass index	2007 to 2011	1.57 (1.50 to1.64)
South Asia	Total cancers	High body-mass index	2012 to 2016	1.81 (1.73 to1.89)
South Asia	Total cancers	High body-mass index	2017 to 2021	2.09 (1.99 to2.19)
Central Asia	Total cancers	High body-mass index	1992 to 1996	1.00 (1.00 to1.00)
Central Asia	Total cancers	High body-mass index	1997 to 2001	0.98 (0.93 to1.04)
Central Asia	Total cancers	High body-mass index	2002 to 2006	0.96 (0.91 to1.03)
Central Asia	Total cancers	High body-mass index	2007 to 2011	0.98 (0.92 to1.04)
Central Asia	Total cancers	High body-mass index	2012 to 2016	1.02 (0.95 to1.09)
Central Asia	Total cancers	High body-mass index	2017 to 2021	1.03 (0.96 to1.10)

Table S11. Cohort effects on mortality of total cancer attributable to high BMI across Asia and five GBD regions.

Location	Cause	Rei	Cohort	Rate ratio
Asia	Total cancers	High body-mass index	1892 to 1901	0.20 (0.16 to 0.25)
Asia	Total cancers	High body-mass index	1897 to 1906	0.25 (0.22 to 0.27)
Asia	Total cancers	High body-mass index	1902 to 1911	0.29 (0.28 to 0.31)
Asia	Total cancers	High body-mass index	1907 to 1916	0.34 (0.33 to 0.36)
Asia	Total cancers	High body-mass index	1912 to 1921	0.38 (0.37 to 0.39)
Asia	Total cancers	High body-mass index	1917 to 1926	0.42 (0.41 to 0.44)
Asia	Total cancers	High body-mass index	1922 to 1931	0.47 (0.46 to 0.48)
Asia	Total cancers	High body-mass index	1927 to 1936	0.51 (0.49 to 0.52)
Asia	Total cancers	High body-mass index	1932 to 1941	0.55 (0.54 to 0.57)
Asia	Total cancers	High body-mass index	1937 to 1946	0.60 (0.58 to 0.61)
Asia	Total cancers	High body-mass index	1942 to 1951	0.66 (0.65 to 0.68)
Asia	Total cancers	High body-mass index	1947 to 1956	0.74 (0.72 to 0.76)
Asia	Total cancers	High body-mass index	1952 to 1961	0.80 (0.78 to 0.82)
Asia	Total cancers	High body-mass index	1957 to 1966	0.86 (0.84 to 0.89)
Asia	Total cancers	High body-mass index	1962 to 1971	0.96 (0.94 to 0.98)
Asia	Total cancers	High body-mass index	1967 to 1976	1.00 (1.00 to 1.00)
Asia	Total cancers	High body-mass index	1972 to 1981	1.03 (1.00 to 1.06)
Asia	Total cancers	High body-mass index	1977 to 1986	1.10 (1.06 to 1.15)
Asia	Total cancers	High body-mass index	1982 to 1991	1.22 (1.16 to 1.28)
Asia	Total cancers	High body-mass index	1987 to 1996	1.19 (1.11 to 1.28)
Asia	Total cancers	High body-mass index	1992 to 2001	1.16 (1.04 to 1.29)
High-income Asia Pacific	Total cancers	High body-mass index	1892 to 1901	0.67 (0.46 to 0.97)

Location	Cause	Rei	Cohort	Rate ratio
High-income Asia Pacific	Total cancers	High body-mass index	1897 to 1906	0.84 (0.70 to 1.01)
High-income Asia Pacific	Total cancers	High body-mass index	1902 to 1911	1.00 (0.87 to 1.14)
High-income Asia Pacific	Total cancers	High body-mass index	1907 to 1916	1.15 (1.01 to 1.30)
High-income Asia Pacific	Total cancers	High body-mass index	1912 to 1921	1.25 (1.11 to 1.41)
High-income Asia Pacific	Total cancers	High body-mass index	1917 to 1926	1.30 (1.16 to 1.46)
High-income Asia Pacific	Total cancers	High body-mass index	1922 to 1931	1.37 (1.22 to 1.54)
High-income Asia Pacific	Total cancers	High body-mass index	1927 to 1936	1.41 (1.25 to 1.58)
High-income Asia Pacific	Total cancers	High body-mass index	1932 to 1941	1.36 (1.21 to 1.53)
High-income Asia Pacific	Total cancers	High body-mass index	1937 to 1946	1.31 (1.17 to 1.46)
High-income Asia Pacific	Total cancers	High body-mass index	1942 to 1951	1.25 (1.12 to 1.40)
High-income Asia Pacific	Total cancers	High body-mass index	1947 to 1956	1.28 (1.15 to 1.43)
High-income Asia Pacific	Total cancers	High body-mass index	1952 to 1961	1.22 (1.09 to 1.36)
High-income Asia Pacific	Total cancers	High body-mass index	1957 to 1966	1.17 (1.04 to 1.31)
High-income Asia Pacific	Total cancers	High body-mass index	1962 to 1971	1.07 (0.95 to 1.21)
High-income Asia Pacific	Total cancers	High body-mass index	1967 to 1976	1.00 (1.00 to 1.00)
High-income Asia Pacific	Total cancers	High body-mass index	1972 to 1981	0.94 (0.79 to 1.11)
High-income Asia Pacific	Total cancers	High body-mass index	1977 to 1986	0.88 (0.69 to 1.12)
High-income Asia Pacific	Total cancers	High body-mass index	1982 to 1991	0.83 (0.58 to 1.17)
High-income Asia Pacific	Total cancers	High body-mass index	1987 to 1996	0.77 (0.47 to 1.28)
High-income Asia Pacific	Total cancers	High body-mass index	1992 to 2001	0.64 (0.27 to 1.50)
East Asia	Total cancers	High body-mass index	1892 to 1901	0.19 (0.08 to 0.42)
East Asia	Total cancers	High body-mass index	1897 to 1906	0.21 (0.16 to 0.27)
East Asia	Total cancers	High body-mass index	1902 to 1911	0.22 (0.20 to 0.25)

Location	Cause	Rei	Cohort	Rate ratio
East Asia	Total cancers	High body-mass index	1907 to 1916	0.24 (0.22 to 0.26)
East Asia	Total cancers	High body-mass index	1912 to 1921	0.28 (0.26 to 0.30)
East Asia	Total cancers	High body-mass index	1917 to 1926	0.32 (0.31 to 0.34)
East Asia	Total cancers	High body-mass index	1922 to 1931	0.38 (0.36 to 0.40)
East Asia	Total cancers	High body-mass index	1927 to 1936	0.43 (0.41 to 0.45)
East Asia	Total cancers	High body-mass index	1932 to 1941	0.48 (0.46 to 0.51)
East Asia	Total cancers	High body-mass index	1937 to 1946	0.54 (0.51 to 0.56)
East Asia	Total cancers	High body-mass index	1942 to 1951	0.62 (0.59 to 0.65)
East Asia	Total cancers	High body-mass index	1947 to 1956	0.70 (0.67 to 0.73)
East Asia	Total cancers	High body-mass index	1952 to 1961	0.77 (0.74 to 0.80)
East Asia	Total cancers	High body-mass index	1957 to 1966	0.82 (0.79 to 0.85)
East Asia	Total cancers	High body-mass index	1962 to 1971	0.93 (0.90 to 0.97)
East Asia	Total cancers	High body-mass index	1967 to 1976	1.00 (1.00 to 1.00)
East Asia	Total cancers	High body-mass index	1972 to 1981	1.10 (1.05 to 1.17)
East Asia	Total cancers	High body-mass index	1977 to 1986	1.26 (1.17 to 1.35)
East Asia	Total cancers	High body-mass index	1982 to 1991	1.40 (1.29 to 1.53)
East Asia	Total cancers	High body-mass index	1987 to 1996	1.44 (1.26 to 1.63)
East Asia	Total cancers	High body-mass index	1992 to 2001	1.46 (1.18 to 1.82)
Southeast Asia	Total cancers	High body-mass index	1892 to 1901	0.12 (0.05 to 0.30)
Southeast Asia	Total cancers	High body-mass index	1897 to 1906	0.16 (0.11 to 0.23)
Southeast Asia	Total cancers	High body-mass index	1902 to 1911	0.20 (0.16 to 0.24)
Southeast Asia	Total cancers	High body-mass index	1907 to 1916	0.23 (0.20 to 0.27)
Southeast Asia	Total cancers	High body-mass index	1912 to 1921	0.26 (0.24 to 0.29)

Location	Cause	Rei	Cohort	Rate ratio
Southeast Asia	Total cancers	High body-mass index	1917 to 1926	0.30 (0.27 to 0.33)
Southeast Asia	Total cancers	High body-mass index	1922 to 1931	0.35 (0.32 to 0.38)
Southeast Asia	Total cancers	High body-mass index	1927 to 1936	0.41 (0.38 to 0.45)
Southeast Asia	Total cancers	High body-mass index	1932 to 1941	0.49 (0.46 to 0.52)
Southeast Asia	Total cancers	High body-mass index	1937 to 1946	0.56 (0.52 to 0.60)
Southeast Asia	Total cancers	High body-mass index	1942 to 1951	0.64 (0.60 to 0.68)
Southeast Asia	Total cancers	High body-mass index	1947 to 1956	0.71 (0.66 to 0.75)
Southeast Asia	Total cancers	High body-mass index	1952 to 1961	0.78 (0.73 to 0.82)
Southeast Asia	Total cancers	High body-mass index	1957 to 1966	0.85 (0.80 to 0.90)
Southeast Asia	Total cancers	High body-mass index	1962 to 1971	0.93 (0.88 to 0.99)
Southeast Asia	Total cancers	High body-mass index	1967 to 1976	1.00 (1.00 to 1.00)
Southeast Asia	Total cancers	High body-mass index	1972 to 1981	1.07 (0.99 to 1.15)
Southeast Asia	Total cancers	High body-mass index	1977 to 1986	1.12 (1.02 to 1.23)
Southeast Asia	Total cancers	High body-mass index	1982 to 1991	1.16 (1.03 to 1.32)
Southeast Asia	Total cancers	High body-mass index	1987 to 1996	1.22 (1.03 to 1.45)
Southeast Asia	Total cancers	High body-mass index	1992 to 2001	1.30 (1.00 to 1.69)
South Asia	Total cancers	High body-mass index	1892 to 1901	0.10 (0.03 to 0.26)
South Asia	Total cancers	High body-mass index	1897 to 1906	0.11 (0.07 to 0.16)
South Asia	Total cancers	High body-mass index	1902 to 1911	0.13 (0.10 to 0.16)
South Asia	Total cancers	High body-mass index	1907 to 1916	0.15 (0.13 to 0.18)
South Asia	Total cancers	High body-mass index	1912 to 1921	0.18 (0.16 to 0.20)
South Asia	Total cancers	High body-mass index	1917 to 1926	0.21 (0.19 to 0.23)
South Asia	Total cancers	High body-mass index	1922 to 1931	0.24 (0.22 to 0.27)

Location	Cause	Rei	Cohort	Rate ratio
South Asia	Total cancers	High body-mass index	1927 to 1936	0.29 (0.26 to 0.31)
South Asia	Total cancers	High body-mass index	1932 to 1941	0.33 (0.31 to 0.36)
South Asia	Total cancers	High body-mass index	1937 to 1946	0.39 (0.36 to 0.42)
South Asia	Total cancers	High body-mass index	1942 to 1951	0.45 (0.42 to 0.49)
South Asia	Total cancers	High body-mass index	1947 to 1956	0.54 (0.49 to 0.58)
South Asia	Total cancers	High body-mass index	1952 to 1961	0.63 (0.58 to 0.68)
South Asia	Total cancers	High body-mass index	1957 to 1966	0.76 (0.70 to 0.82)
South Asia	Total cancers	High body-mass index	1962 to 1971	0.86 (0.79 to 0.93)
South Asia	Total cancers	High body-mass index	1967 to 1976	1.00 (1.00 to 1.00)
South Asia	Total cancers	High body-mass index	1972 to 1981	1.13 (1.02 to 1.25)
South Asia	Total cancers	High body-mass index	1977 to 1986	1.17 (1.04 to 1.31)
South Asia	Total cancers	High body-mass index	1982 to 1991	1.24 (1.08 to 1.43)
South Asia	Total cancers	High body-mass index	1987 to 1996	1.28 (1.08 to 1.51)
South Asia	Total cancers	High body-mass index	1992 to 2001	1.26 (1.00 to 1.60)
Central Asia	Total cancers	High body-mass index	1892 to 1901	0.91 (0.35 to 2.38)
Central Asia	Total cancers	High body-mass index	1897 to 1906	0.75 (0.47 to 1.19)
Central Asia	Total cancers	High body-mass index	1902 to 1911	0.70 (0.51 to 0.95)
Central Asia	Total cancers	High body-mass index	1907 to 1916	0.76 (0.61 to 0.95)
Central Asia	Total cancers	High body-mass index	1912 to 1921	0.83 (0.69 to 1.00)
Central Asia	Total cancers	High body-mass index	1917 to 1926	0.97 (0.82 to 1.14)
Central Asia	Total cancers	High body-mass index	1922 to 1931	1.04 (0.89 to 1.21)
Central Asia	Total cancers	High body-mass index	1927 to 1936	1.06 (0.91 to 1.23)
Central Asia	Total cancers	High body-mass index	1932 to 1941	1.10 (0.95 to 1.27)

Location	Cause	Rei	Cohort	Rate ratio
Central Asia	Total cancers	High body-mass index	1937 to 1946	1.10 (0.95 to 1.27)
Central Asia	Total cancers	High body-mass index	1942 to 1951	1.12 (0.97 to 1.29)
Central Asia	Total cancers	High body-mass index	1947 to 1956	1.09 (0.95 to 1.26)
Central Asia	Total cancers	High body-mass index	1952 to 1961	1.04 (0.91 to 1.20)
Central Asia	Total cancers	High body-mass index	1957 to 1966	1.00 (0.87 to 1.15)
Central Asia	Total cancers	High body-mass index	1962 to 1971	0.97 (0.84 to 1.12)
Central Asia	Total cancers	High body-mass index	1967 to 1976	1.00 (1.00 to 1.00)
Central Asia	Total cancers	High body-mass index	1972 to 1981	0.99 (0.81 to 1.20)
Central Asia	Total cancers	High body-mass index	1977 to 1986	0.94 (0.74 to 1.19)
Central Asia	Total cancers	High body-mass index	1982 to 1991	0.90 (0.67 to 1.20)
Central Asia	Total cancers	High body-mass index	1987 to 1996	0.86 (0.59 to 1.27)
Central Asia	Total cancers	High body-mass index	1992 to 2001	0.93 (0.50 to 1.72)

Figure S1. Deaths of total cancer attributable to high BMI in 2021 across 34 Asian countries and territories.

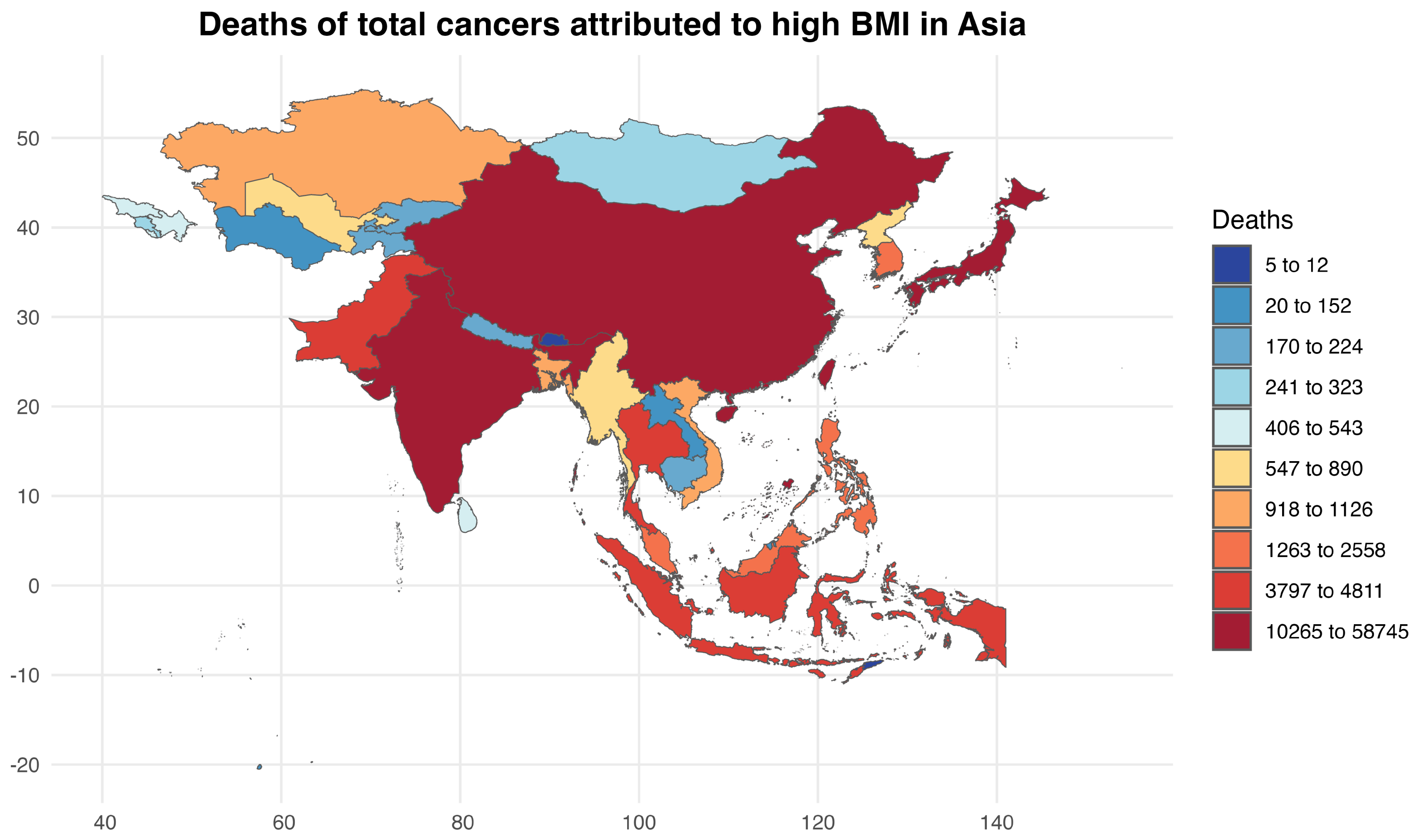

Figure S2. ASMR of total cancer attributable to high BMI in 2021 across 34 Asian countries and territories.

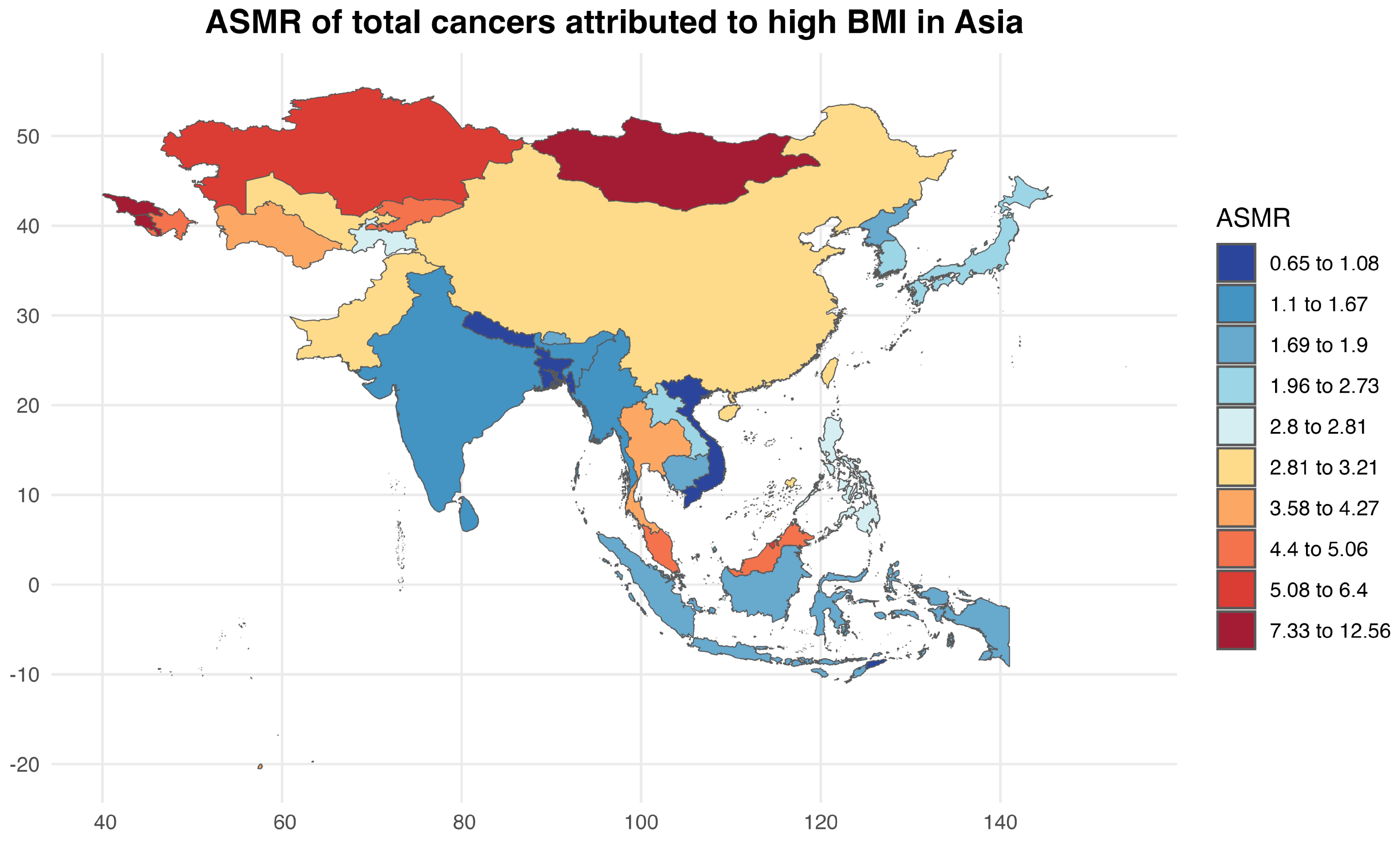

Figure S3. AAPC of ASMR for total cancer attributable to high BMI from 1992 to 2021 across 34 Asian countries and territories.

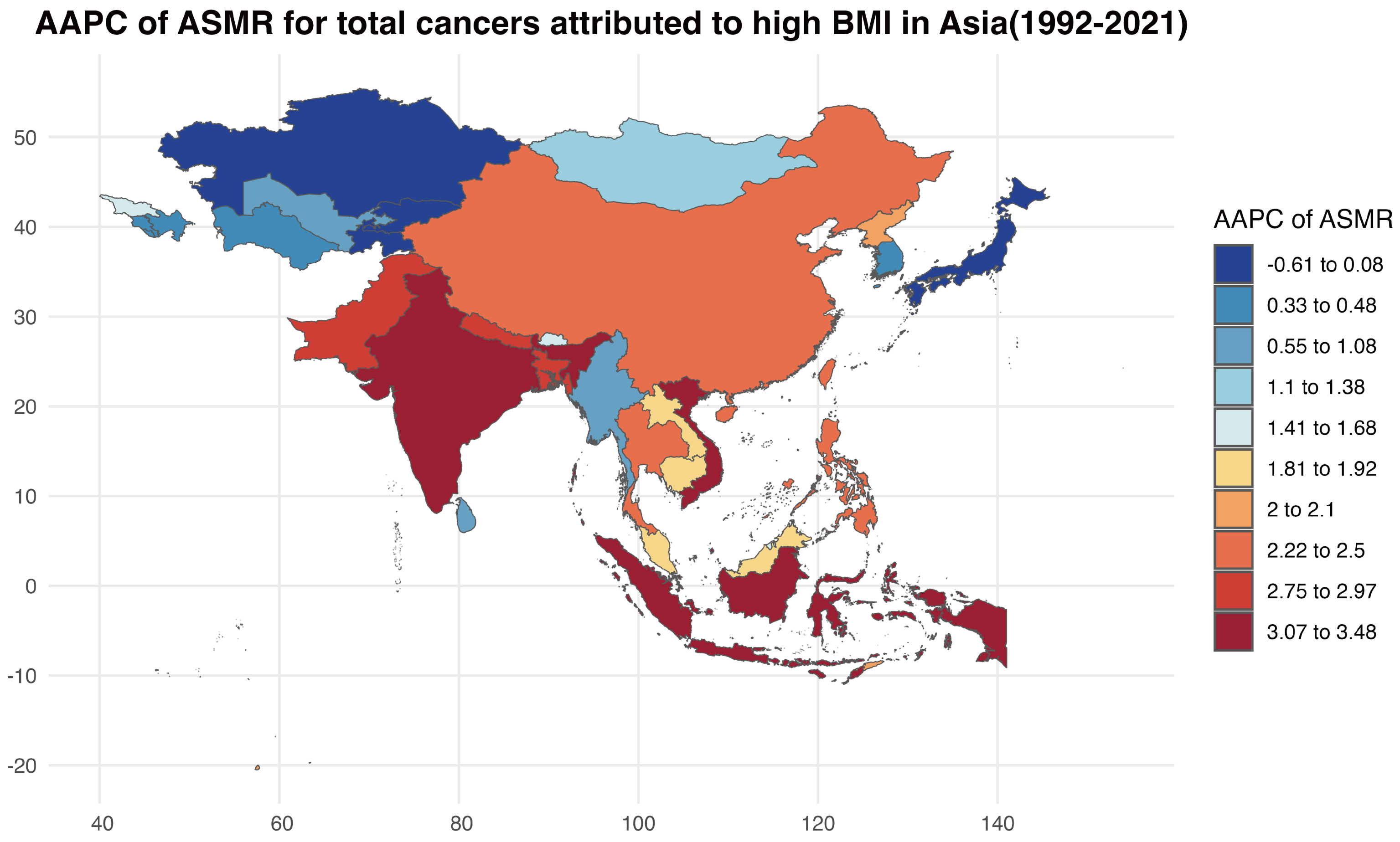

Supplement: Supplementary file 1 [file DataSheet1.pdf]
